# Modulating TERRA G-quadruplexes with ligands: impact on telomeric DNA:RNA hybrids and ALT mechanisms

**DOI:** 10.1093/nar/gkaf1300

**Published:** 2025-11-29

**Authors:** Federico Dinoi, Simona Marzano, Maria Ilaria Marino, Eleonora Vertecchi, Carlo Maria D’Angelo, Carmen Maresca, Eleonora Petti, Roberto Dinami, Angela Rizzo, Annamaria Biroccio, Stefano Cacchione, Bruno Pagano, Erica Salvati, Jussara Amato

**Affiliations:** Institute of Molecular Biology and Pathology, National Research Council, Rome 00185, Italy; Department of Biology and Biotechnology “Charles Darwin”, Sapienza University of Rome, Rome 00185, Italy; Department of Pharmacy, University of Naples Federico II, Naples 80131, Italy; Institute of Molecular Biology and Pathology, National Research Council, Rome 00185, Italy; Institute of Molecular Biology and Pathology, National Research Council, Rome 00185, Italy; Institute of Molecular Biology and Pathology, National Research Council, Rome 00185, Italy; Translational Oncology Research Unit, IRCCS-Regina Elena National Cancer Institute, Rome 00144, Italy; Translational Oncology Research Unit, IRCCS-Regina Elena National Cancer Institute, Rome 00144, Italy; Translational Oncology Research Unit, IRCCS-Regina Elena National Cancer Institute, Rome 00144, Italy; Translational Oncology Research Unit, IRCCS-Regina Elena National Cancer Institute, Rome 00144, Italy; Translational Oncology Research Unit, IRCCS-Regina Elena National Cancer Institute, Rome 00144, Italy; Department of Biology and Biotechnology “Charles Darwin”, Sapienza University of Rome, Rome 00185, Italy; Department of Pharmacy, University of Naples Federico II, Naples 80131, Italy; Institute of Molecular Biology and Pathology, National Research Council, Rome 00185, Italy; Department of Pharmacy, University of Naples Federico II, Naples 80131, Italy

## Abstract

Telomeres are transcribed into the long non-coding RNA TERRA, which is essential for telomere protection and maintenance. In cancer cells, telomere lengthening occurs via telomerase reactivation or the alternative lengthening of telomeres (ALT). TERRA is highly overexpressed in ALT cells and directly influences this process. However, due to the lack of efficient tools to investigate TERRA biology, its role in cancer progression and its potential as a therapeutic target remains unclear. Both telomeric DNA and TERRA form noncanonical structures called G-quadruplexes (GQs) on their G-rich strands, which can be the targets of GQ ligands. Using a ligand-based virtual screening of FDA-approved drugs, we identified novel TERRA GQ ligands capable of stabilizing TERRA binding to chromatin. This interaction increased telomeric DNA:RNA hybrids, induced telomeric defects, and elevated ALT-associated PML bodies formation in both telomerase- and ALT-positive cancer cells in an RNAseH1 dependent manner. These ligands also partly increased C-circle levels. *In vitro*, these ligands recognized and stabilized DNA:RNA GQ hybrids, revealing a novel mechanism of TERRA binding to telomeric DNA, which may contribute to replication stress, sister-telomere disjunction impairment, and enhanced ALT activity, offering new insights into TERRA’s multifaceted role in telomere dynamics and its implications for cancer biology.

## Graphical Abstract

**Graphical Abstract:**
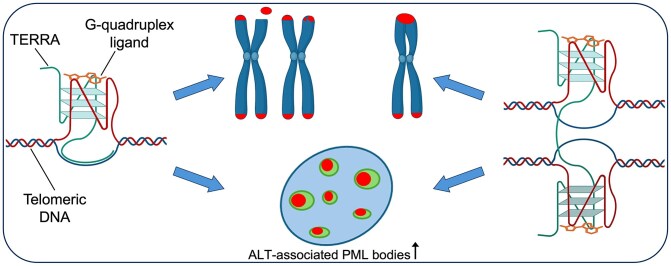

## Introduction

Telomeres are protective nucleoprotein structures located at the ends of eucaryotic chromosomes, characterized by tandem repeats of G-rich sequences. The architecture of mammalian telomeric chromatin is unique: it contains both nucleosomes and telomere-specific protein complexes (shelterin and CTC1–STN1–TEN1 complexes), which are responsible for the suppression of the DNA damage response pathway and for maintaining telomere homeostasis [1]. Telomeric DNA is transcribed into a G-rich long non-coding RNA known as TERRA (telomeric repeat-containing RNA), consisting of several UUAGGG repeats, with promoters situated in the subtelomeres of all chromosomes [2, 3]. TERRA associates with telomeric DNA forming DNA:RNA hybrids, both in *cis* (co-transcriptionally) and in *trans*, through sequence complementarity with the C-rich strand [4]. These DNA:RNA hybrids, along with the displaced G-rich DNA strand, lead to the formation of a loop structure called the R-loop. This structure strongly impacts telomeric chromatin topology and consequently telomere stability [5–7]. TERRA is implicated in various processes, including telomere protection, telomerase regulation, and epigenetic modifications of telomeric chromatin [8–11]. In normal cells, TERRA expression is inversely correlated with telomere length since it is a negative regulator of telomerase [6, 12], whereas, in human cancers, the relationship between TERRA expression and cancer progression remains poorly understood. Most human cancers reactivate telomerase to sustain telomere length and achieve replicative immortality, while in a small subset of cancers (10%–15%), telomeres are maintained through an alternative mechanism involving homologous recombination and break-induced DNA replication, known as ALT (alternative lengthening of telomeres) [13, 14]. Notably, TERRA is highly expressed in ALT-positive cancer cells and is proposed to play a key role in initiating the ALT mechanism [5, 15] Supporting this, TERRA downregulation has been shown to impair the ALT pathway, disrupt telomere maintenance, and ultimately lead to cell replication arrest [16]. The elusive role of TERRA in cancer initiation and progression is partly due to lack of specific interactors and effective tools to modulate its expression or association with telomeric chromatin. Addressing these gaps is critical for unraveling TERRA’s contribution to telomere biology and its potential as a therapeutic target.

Given the presence of several G-runs, TERRA readily folds into G-quadruplex (GQ) structures, composed by stacked G-quartets, which are planar arrangement of four guanine bases stabilized by Hoogsteen hydrogen bonds, and further stabilized by the presence of monovalent cations such as potassium or sodium [17]. GQs can adopt various topologies, including parallel, antiparallel, or hybrid [3+1], depending on strand orientation and loop configuration. TERRA GQ structures have been shown to play a central role in TERRA biology, facilitating interactions with TERRA RNA with telomeric proteins TRF1 and TRF2 [18]. In addition, TERRA hybridization with telomeric DNA induces GQ formation on the displaced G-rich strand, whose stabilization has been linked to enhanced ALT activity. This is supported by studies showing that GQ stabilizers increase ALT activity [19–21].

TERRA GQs exhibit predominantly a parallel topology [22], making them promising targets for specific ligands. In a previous work, we identified a TERRA GQ ligand capable of inducing telomeric DNA damage and antiproliferative effects in ALT cells, even at low concentrations [23]. In this study, we conducted a ligand-based virtual screening (LBVS) of FDA-approved drugs to find new compounds that interact with RNA GQs [24]. These compounds were experimentally validated and prioritized based on their ability to bind TERRA GQ. Using these validated compounds, we explored the molecular events triggered by TERRA GQ stabilization in both telomerase- and ALT-positive cancer cells. Our findings offer deeper insights into the intricate mechanism of TERRA binding to telomeric chromatin. Noteworthy, this work advances our understanding of how TERRA stabilization could impact telomere maintenance, genome stability, and survival of both telomerase- and ALT-positive cancer cells, underscoring the potential therapeutic implications of TERRA targeting in cancer therapy.

## Material and methods

### Cell culture, treatments, transfection, and reagents

Human cervical cancer cells (HeLa) and human osteosarcoma cells (U2OS) were purchased from ATCC repository. Cells were maintained in Dulbecco Modified Eagle Medium supplemented with 10% fetal calf serum, 2 mM l-glutamine, and antibiotics. For transfection experiments, U2OS cells were seeded at 30% confluency and transfected the following day with pAIO hM27RNaseH1-EGFP wild type and pAIO hM27RNaseH1-EGFP D210 constructs [25], or with RHINO plasmid (kindly provided by Prof. De Almeida) [26] with jetPEI reagent (Euroclone) according to the manufacturer’s instructions. For treatments, cells were seeded at 30%–40% confluency, and the day after were administered with freshly prepared dilutions of compounds or vehicle (dimethyl sulfoxide (DMSO).

CPG supports, (2′-OTBDMS)-RNA and DNA phosphoramidites, as well as all reagents for oligonucleotide synthesis were purchased from Link Technologies (Bellshill, UK). All other reagents and solvents were purchased from Merck KGaA (Darmstadt, Germany). Sensor chips, amino coupling reagents, and buffers for surface plasmon resonance (SPR) measurements were purchased from GE Healthcare. All buffers were prepared with highly purified Milli-Q water and sterilized before use by autoclaving and/or the addition of diethylpyrocarbonate (DEPC, Merck). Bazedoxifene, berbamine, bisoctrizole, nilotinib, quercetin, and thiazole orange (TO) were purchased from Merck. Cepharantine, ceritinib, enzastaurin, fedratinib, ledipasvir, netarsudil, novobiocin, osimertinib, pyridostatin (PDS), and pranlukast were purchased from DivBioScience (Ulvenhout, Netherlands). Duvelisib was provided by CliniSciences (Guidonia Montecielo, Italy).

### Oligonucleotide synthesis and sample preparation

TERRA (5′-*UAGGGUUAGGGU*-3′), Tel26 (5′-TTAGGGTTAGGGTTAGGGTTAGGGTT-3′), and the duplex-forming ds26 (5′-CAATCGGATCGAATTCGATCCGATTG-3′) sequences were synthesized using standard β-cyanoethyl phosphoramidite solid phase chemistry on an ABI 394 DNA/RNA synthesizer (Applied Biosystem, Foster City, CA, USA) at 1 µM scale. As for RNA synthesis, 5-benzylthio-1-H-tetrazole instead of 4,5-dicyanoimidazole was used as activator reagent, and coupling steps were prolonged of 5 min. A concentrated NH_4_OH/EtOH (3:1, v/v) solution was used to deprotect and detach RNA at room temperature (r.t.) for 12 h. For DNA sequences, deprotection and detachment were performed by using a concentrated NH_4_OH aqueous solution at 55°C for 12 h. Both RNA and DNA sequences were purified by high-performance liquid chromatography on a Nucleogel SAX column (1000-8/46, Macherey-Nagel, GmbH & Co. KG, Dueren, Germany) using a 30 min linear gradient going from 100% buffer A [20 mM KH_2_PO_4_/K_2_HPO_4_ buffer, pH 7.0, containing 20% (v/v) CH_3_CN] to 100% buffer B [20 mM KH_2_PO_4_/K_2_HPO_4_ buffer, pH 7.0, containing 1.0 M KCl and 20% (v/v) CH_3_CN] with a flow rate of 1 ml/min [27]. The fractions of the oligomers were collected and successively desalted by Sep-Pak cartridges (C-18). Lastly, 2′-TBDMS group in RNA was removed by Et_3_N·3HF/DMF (1:3, v/v) at r.t. for 12 h. The reaction was quenched with 0.1 M TEAA buffer (pH 7.0), and the deprotected RNAs were again desalted on a Sep-pak (C-18) cartridge. All oligonucleotides were proven to be >98% pure by nuclear magnetic resonance (NMR). The chimeric DNA–RNA oligonucleotide HGQ24 forming a DNA:RNA hybrid GQ (5′-TAGGGTTAGGG*UUAGGGUUAGGGU*-3′) (RNA residues are in italics) was provided from Biomers (Ulm, Germany) and used without further purification. All oligonucleotide samples were prepared in a 5 mM KH_2_PO_4_/K_2_HPO_4_ (phosphate) buffer (pH 7.0), supplemented with 20 mM KCl, and their concentration was measured by UV adsorption at 90°C using the appropriate molar extinction coefficient values (ε) at λ = 260 nm, calculated by the nearest-neighbor model [28]. Samples were then annealed by heating at 90°C for 5 min, followed by a slow cooling to room temperature overnight, and stored at 4°C for 24 h before measurements.

### Fluorescent intercalator displacement assay with thiazole orange

Measurements were performed using an FP-8300 spectrofluorometer (Jasco, Easton, MD, USA) equipped with a Peltier cell holder (Jasco PCT-818), in a 1-cm path-length cell at 20°C. A solution containing 0.25 µM of each prefolded GQ target—such as HGQ24, TERRA, or Tel26—and 0.5 µM of TO in 5 mM phosphate buffer (pH 7.0) supplemented with 20 mM KCl was prepared. Fluorescence spectra were recorded in the absence and presence of increasing concentrations of drugs (10 mM stock solution in pure DMSO). After drug addition, a 3-min equilibration time was allowed before acquiring the spectrum. Measurements were performed using an excitation wavelength of 485 nm and an emission range of 500–650 nm, with both excitation and emission slits set at 5 nm. The percentage of TO displacement was calculated using the formula: TO displacement (%) = 100 − [(*F*/*F*0) × 100], where *F*0 represents the fluorescence in the absence of the drug and *F* is the fluorescence after each drug addition. The percentage of displacement was then plotted as a function of drug concentration, and the DC_50_ value (the concentration required to displace 50% of TO from the nucleic acid structures) was determined. Each titration was performed in triplicate.

### Circular dichroism experiments

Circular dichroism (CD) experiments were performed using a Jasco J-815 spectropolarimeter (Jasco, Easton, MD, USA) equipped with a PTC-423S/15 Peltier temperature controller. Oligonucleotide samples, prepared at a GQ concentration of 2 µM, were placed in a 1-cm path-length cell for measurements. Oligonucleotide/drug mixtures were recorded by adding 10 mol equiv of ligand (with drug stock solutions of 10 mM in DMSO). CD spectra were recorded at 20°C and 100°C with a scan rate of 100 nm/min, a 2-s response time, and a 2-nm bandwidth, over a wavelength range of 230–360 nm. The results were averaged over three scans and baseline-corrected by subtracting the spectrum of the buffer alone. CD melting experiments were carried out over a temperature range of 20°C–100°C, with a heating rate of 1°C/min, by monitoring changes in the CD signal at the wavelengths of maximal CD intensity (264 nm for HGQ24 and TERRA, and 290 nm for Tel26), in the absence and presence of ligands (10 mol equiv) [29]. The melting temperatures (*T*_m_) of GQs were determined from a curve fit using OriginPro 2021 software (OriginLab Corp., Northampton, MA, USA). ∆*T*_m_ values were determined as the difference in the *T*_m_ values of the nucleic acid structures in the presence and absence of ligands. All experiments were performed in triplicate, and the reported values represent the average of the three measurements.

### Nondenaturing polyacrylamide gel electrophoresis

Nondenaturing 20% polyacrylamide gel electrophoresis (PAGE) gels were prepared using a 29:1 acrylamide/bisacrylamide solution and 1× TBE (Tris-Borate-EDTA) buffer. Gels were run in 1× TBE buffer on a Mini-PROTEAN electrophoresis system (Bio-Rad, CA, USA) at 4°C, 120 V for 2 h [30]. Oligonucleotide samples were prepared at a GQ concentration of 20 μM in 5 mM phosphate buffer (pH 7.0) supplemented with 20 mM KCl. To facilitate sample loading in the wells 10% glycerol was added. Bands were visualized by UV shadowing at 254 nm.

### Fluorescence titrations

Fluorescence titration experiments were performed at 25°C using a Jasco FP-8300 spectrofluorometer (Jasco, Easton, MD, USA) equipped with a Peltier temperature controller (PCT-818) and a sealed quartz cuvette (1 cm path-length). Fluorophore-labeled GQ-forming sequences were used to obtain the values of apparent equilibrium dissociation constants (*K*_d_), exploiting the ligand-induced dose-dependent quenching of fluorophore’s emission [31, 32]. Labeled oligonucleotides [(Cy5.5–5′-TAGGGTTAGGG*UUAGGGUUAGGGU*-3′) Cy5.5-HGQ24 and (Cy5.5–5′-TTAGGGTTAGGGTTAGGGTTAGGGTT-3′) Cy5.5-Tel26] were obtained from Biomers (Ulm, Germany), used without further purification, and annealed as described above before use. Titrations were performed by stepwise additions of drugs (0.25–20 µl, from ligand stock solutions in pure DMSO at concentration of 0.2 mM for netarsudil, 1 mM for osimertinib, and 2 mM for fedratinib) to the labeled GQ structures (0.2 µM) in 5 mM phosphate buffer (pH 7.0) supplemented with 20 mM KCl. Excitation wavelength was set to 675 nm and emission spectra were recorded over the range 685–750 nm. Both excitation and emission slit widths were set to 10 nm. Following each drug addition, the solutions were gently stirred and allowed to equilibrate for 3 min before recording the spectra. *K*_d_ values were determined by plotting ∆*F/*∆*F*_max_ at 694 nm versus drug concentration and fitting the data using the Hill saturation model for one-site binding: $\frac{{\Delta F}}{{\Delta {{F}_{{\rm max}}}}} = {{B}_{{\rm max}}}[ {\mathit{\mathrm{drug}}} ]/( {{{K}_d} + [ {\mathit{\mathrm{drug}}} ]} )$, where ∆*F* is the change in the maximum fluorescence intensity of the labeled oligonucleotide after each drug addition, ∆*F*_max_ is the fluorescence change at saturation, *B*_max_ represents the maximum specific binding (in the same units as ∆*F/*∆*F*_max_), and *K*_d_ is the dissociation constant [33, 34]. OriginPro 2021 software (OriginLab Corp., Northampton, MA, USA) was used to plot and analyze the binding curves. Experiments were performed in duplicate, and the reported *K*_d_ values represent the mean ± standard deviation of the two measurements.

### Förster resonance energy transfer melting experiments

Förster resonance energy transfer (FRET) experiments were performed using a Jasco FP-8300 spectrofluorometer equipped with a Peltier temperature controller (PCT-818) and a sealed quartz cuvette (1 cm path-length). A dual-labeled FAM/TAMRA GQ-forming telomeric DNA sequence FAM-5′-GGGTTAGGGTTAGGGTTAGGG-3′-TAMRA (F-Tel21-T) was used [35]. The oligonucleotide was first dissolved in water at 1 mM, diluted to 1 μM using 5 mM phosphate buffer (pH 7.0) supplemented with 20 mM KCl and annealed by heating to 90°C for 5 min, followed by cooling to room temperature overnight. Experiments were carried out using 0.2 μM of F-Tel21-T GQ, 2 μM of ligand, and 0, 5, or 10 μM (final concentration) of the DNA duplex competitor ds26 [36]. A blank sample without ligand and competitor was also included. Fluorescence spectra were acquired both before (at 20°C) and after (at 95°C) the melting assay. The dual-labeled oligonucleotide was excited at 492 nm, and emission spectra were recorded between 500–650 nm. Excitation and emission slit widths were set at 5 nm. FRET melting was monitored by following the emission intensity of FAM at 520 nm (upon excitation at 492 nm), using a temperature ramp of 0.2°C/min from 20°C to 95°C. Emission of FAM was normalized between 0 and 1. Final data analysis was performed using OriginPro 2021 software.

### Surface plasmon resonance experiments

SPR experiments were performed at 25°C using a Biacore X100 (GE Healthcare) equipped with a research-grade CM5 sensor chip. The anti-DNA/RNA GQ antibody (BG4) (#MaBe917, Merck-Millipore, Prague, Czech Republic) was immobilized via amine-coupling chemistry, with HBS-EP as the running buffer (10 mM HEPES buffer, pH 7.4, containing 150 mM NaCl, 3 mM EDTA, and 0.005% surfactant P20) [37]. The flow cell surfaces were activated with a 1:1 mixture of 0.1 M N-hydroxysuccinimide and 0.1  M 3-(N,N-dimethylamino)propyl-N-ethylcarbodiimide at a flow rate of 10 μl/min. BG4, diluted to a concentration of 50 μg/ml in 10 mM sodium acetate (pH 4.5), was immobilized at a density of 2500 RU on the sample flow cell, while the reference cell remained blank. Unreacted activated groups were blocked by the injection of 1.0 M ethanolamine at 10 μl/min over the chip surface. HGQ24, TERRA, and Tel26 GQs were prepared at a concentration of 20 µM and then diluted at 1 µM. Kinetic binding data were collected by using the single-cycle kinetics approach [38]. Oligonucleotides were injected sequentially in the same cycle, from low to high concentrations (ranging from 0.06 to 1 μM), with an association time of 30 s and a dissociation time of 600 s at the end. Injections were performed at a flow rate of 30 μl/min using a running solution of 5 mM phosphate buffer at pH 6.0, supplemented with 20 mM KCl. No regeneration was required after each sample. Data were fitted to a simple 1:1 interaction model using the global data analysis option available within the BIAevaluation software provided with the device.

### IF and IF/DNA–FISH on interphase cells

At the end of treatments, cells were fixed in 2% formaldehyde and permeabilized in 0.25% Triton X-100 in PBS for 5 min at room temperature at each endpoint. For anti-R-loop staining only, cells were fixed and permeabilized with methanol:acetone solution 1:1 for 10 min at −20°C and treated with RNAseH1 (New England Biolabs) 50U/slide for 6 h at 37°C or left untreated. Finally, samples were processed for immunolabeling with anti-PML (PG-M3 Mouse SC-966 and N-19 Goat SC-9862, Santa Cruz Biotechnologies) or anti-DNA:RNA hybrids (Clone S9.6 Mouse MABE 1095, Merk Millipore) antibodies, followed by the anti-mouse IgG or anti-rabbit IgG Alexa fluor 488 secondary antibodies (Cell Signaling). RHINO-transfected cells were processed for immunostaining against TRF2 using the monoclonal mouse anti-TRF2 antibody (clone 4A794 Merck Millipore) followed by a Goat anti-Mouse Alexa Fluor 555 secondary antibody (Cell Signalling). Then, samples were refixed in 2% formaldehyde, dehydrated with ethanol series (70%, 90%, 100%), air dried, co-denatured for 3 min at 80°C with a Cy3-labeled PNA probe, specific for telomere sequences (TelC-Cy3, Panagene, Daejon, South Korea), and incubated for 2 h in a humidified chamber at room temperature in the dark. After hybridization, slides were washed with 70% formamide, 10 mM Tris–HCl (pH 7.2), BSA 0.1%, and then rinsed in TBS/Tween 0.08%, dehydrated with ethanol series, and finally counterstained with DAPI (0.5 μg/ml; Sigma–Aldrich) and mounted on slides in mounting medium (Gelvatol Moviol, Sigma Aldrich). Fluorescence signals were acquired with a CrestOptics V3 confocal spinning disk mounted on a Nikon Ti2-E Inverted microscope with an integrated camera and a laser source (Lumencor).

### Telo-FISH on metaphase spreads

For telomere doublets analysis, chromosome spreads were obtained following 4-h incubation in colchicine 5 μM (Sigma–Aldrich) and prepared following standard procedure consisting of treatment with a hypotonic solution (75 mM KCl) for 20 min at 37°C, followed by fixation in freshly prepared Carnoy solution [3:1 (v/v) methanol/acetic acid]. Cells were then dropped onto slides, air dried, and utilized for cytogenetic analysis. Staining of centromeres and telomeres was performed as previously described [39] using the TelC-Cy3 PNA probe, and an Alexa488-labeled PNA probe specific for the human alphoid DNA sequence to mark centromeres (Cent-Alexa488) (both from Panagene, Daejon, South Korea). Metaphase images were captured using an Olympus AX70 fluorescence microscope at 100× magnification.

### IF/RNA–FISH

The RNA FISH probe was prepared as follows: 1 µg of telomeric dsDNA of 1.6-kb length was denatured at 95°C for 5 min and renatured in ice for 5 min in presence of 200 pmol of a ssDNA (CCCTAA)_7_ in TE buffer to obtain the G-rich single strand. The oligo was labeled with Cy3-dCTP with the Random Prime Labelling System (GE Healthcare) overnight, purified with Microspin G-25 Columns (GE Healthcare), and precipitated with 40 µg of yeast tRNA at −20°C overnight. The pellet was resuspended in RNA–FISH oligo Hyb solution (10% dextrane sulfate, 2XSSC, and 50% formamide) and denaturated for 10 min at 72°C and 10 min at 60°C. Cells were seeded on top of coverslips and treated as above. At the end of treatments, cells were rinsed in ice-cooled cytobuffer (100 mM di NaCl, 300 mM saccarose, 3 mM MgCl_2_, and 10 mM Pipes pH 6.8), permeabilized with cytobuffer with Triton X-100 0.5%, and fixed in 2% formaldehyde. Then cells were blocked in 0.5 M EDTA, 0.8 U/µl RNAse inibitor, 0.5% blocking reagent (Roche) for 1 h at 4°C and incubated with the monoclonal mouse anti-TRF2 antibody (clone 4A794 Merck Millipore) followed by a Goat anti-Mouse Alexa Fluor 488 secondary antibody (Cell Signalling). Successively, cells were refixed in 2% formaldehyde for 10 min at r.t. and dehydrated with ethanol series (70%, 90%, 100%). Dehydrated samples were incubated with the probe prepared as above or with the Cy3-labeled PNA probe (TelC-Cy3, Panagene, Daejon, South Korea) at r.t. overnight. Finally, coverslips were rinsed in 2XSSC and PBS, counterstained with DAPI (0.5 μg/ml, Sigma–Aldrich) and mounted on slides in mounting medium (Gelvatol Moviol, Sigma–Aldrich). Fluorescence signals were acquired by a Nikon Crest Spinning disk at 60× magnitude and analyzed with NIS software.

### Real-time qPCR

Real-time qPCR analysis of TERRA was performed as described in [40]. Briefly, RNA was extracted from cells with RNeasy mini kit (Qiagen) and accurately digested with the RNase-free DNase set (Qiagen). Then, RNA quality was checked on FA gel electrophoresis, retro-transcribed with Superscript IV (Thermo), and amplified in real-time PCR assay with subtelomere-specific primers with a 7900 HT Fast Real-Time PCR System (Applied Biosystem, Waltham, MA, USA).

Primer list:

10q TERRA—forward AAAGCGGGAAACGAAAAGC

10q TERRA—reverse GCCTTGCCTTGGGAGAATCT

XqYq TERRA—forward GAAAGCAAAAGCCCCTCTGA

XqYq TERRA—reverse CCCCTTGCCTTGGGAGAA

XpYp TERRA—forward GCGCGTCCGGAGTTTG

XpYp TERRA—reverse CCACAACCCCACCAGAAAGA

### C-circle assay

C-circle assay was performed as described in [41]. Total DNA was extracted using Quick C-Circle Lysis buffer (50 mM KCl, 10 mM Tris–HCl, pH 8.5, 2 mM MgCl2, 0.5% NP40, 0.5% Tween 20) and treated with 0.5 µg/µl protease (Qiagen, Hilden, Germany). DNA concentration of samples was determined by fluorimetric quantification, and 20 ng of DNA were combined with 10 μl of Φ29 master mix (0.2 mg/ml BSA, 10% Tween, 100 mM each dATP, dGTP, and dTTP, 1× Φ29 buffer), with or without 0,5 U Φ29 DNA polymerase (NEB) and incubated at 30°C for 4 h, then 70°C for 20 min. For quantification, the reaction products were dot-blotted onto a 2× SSC-soaked nylon positively charged membrane (GE Healthcare UK Limited, Little Chalfont, UK). DNA was UV-cross-linked onto the membrane, which was then hybridized with ^32^P-labeled probe (T_2_AG_3_) in Church buffer (0.5 M phosphate buffer, pH 7.2, 7% SDS, 1 mM EDTA, 0.1% BSA) overnight at 55°C. The gel was washed twice and exposed to a PhosphorImager screen and analyzed by Quantity One software (Biorad, Hercules, CA, USA). Signals of samples without Φ29 DNA polymerase were subtracted from signal obtained from corresponding samples with Φ29 DNA polymerase to determine the C-circle expression value.

### TERRA northern blot

RNA (20 μg/sample) was extracted from cell pellets with GENEZOL TriRNA pure kit (Geneaid Biotech Ltd., Taiwan), denatured in 2.3× Denaturant (2.5 ml 40 × MOPS, 2.5 ml H2O, 35 ml formaldehyde, 100 ml deionized formamide) for 15 min at 65°C, and separated by 1.2% agarose formamide gel in 1× Mops buffer at 120 V. Electrophoresis was stopped when bromophenol blue dye reached 8 cm from wells. After electrophoresis, RNA samples were transferred on nylon positively charged membrane (GE Healthcare UK Limited, Little Chalfont, UK) with 20× SSC overnight and UV-cross-linked onto membrane at 125 mJ in UV cross-linker (Hoefer, Holliston, MA, USA). For RNA detection, the blot was hybridized with a ^32^P-labeled probe (T_3_AG_2_) in Church buffer (0.5 M phosphate buffer, pH 7.2, 7% SDS, 1 mM EDTA, 0.1% BSA) overnight at 55°C. The gel was washed twice and exposed to a PhosphorImager screen and analyzed by Quantity One software (Biorad, Hercules, CA, USA).

## Results

### Experimental validation of the ability of drugs identified by virtual screening to bind to TERRA GQ

Following the principle that small molecules with similar structural and geometric features are likely to exhibit comparable binding properties, some of us recently employed a LBVS strategy to identify FDA-approved drugs with potential as RNA GQ ligands [24]. This approach, previously successful in identifying drugs that bind and stabilize SARS-CoV-2 GQ-forming RNA, involved selecting active RNA GQ ligands from the literature and building 3D pharmacophore models based on their structures. These models were then employed in the LBVS of about 3000 FDA-approved drugs, leveraging drug repurposing principles to uncover new therapeutic potentials, identifying 15 drugs as potential RNA GQ ligands [24].

**Figure 1.**
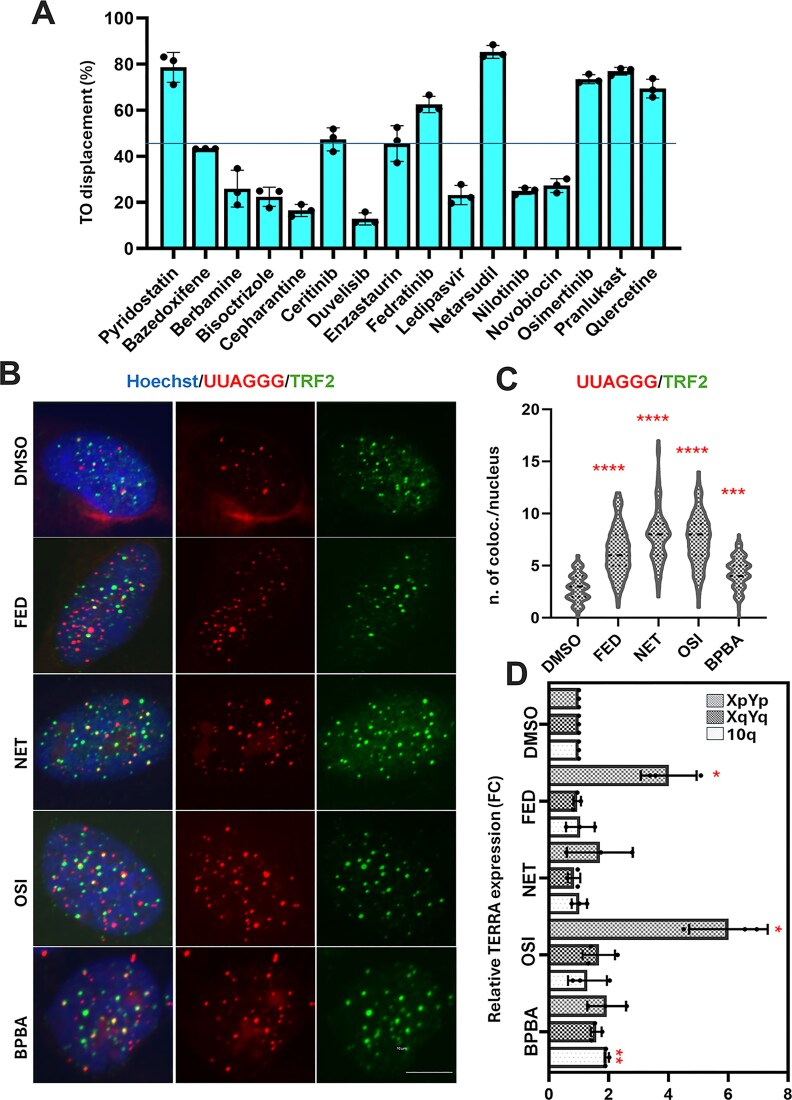
Evaluation of drugs binding to TERRA GQ *in vitro* and their effects on TERRA localization at telomeres. (**A**) Percentage of TO displacement from TERRA GQ upon addition of 12.5 μM of each drug. (**B**) Representative images of U2OS treated with the IC_50_ concentrations of the indicated compounds, stained with an anti-TRF2 antibody to mark telomeres, followed by TERRA RNA FISH hybridization, and finally counterstained with Hoechst to mark nuclei (60× magnification). Images represent the Maximum Intensity Projection (MIP) of 60–80 µm Z-stacks (z. step 0.60 µm). (**C**) Quantitative analysis of the mean number of colocalization between TRF2 spots and TERRA spots per nucleus. Two experiments have been performed and pooled. *N* > 60. Statistical significance was calculated using the Kruskal–Wallis test. *P > .05; ***P > .001; ^****^P > .0001. (**D**) Quantitative analysis of relative TERRA expression in treated and untreated cells. The histograms represent the average number of four independent experiments plotted in the graph as individual points, with bars indicating standard deviation (SD). Statistical significance was calculated using the paired *t*-test (**P < .01).

In this study, the selected molecules were first evaluated *in vitro* for their ability to bind to TERRA GQ. Binding ability was assessed using the well-established fluorescent intercalator displacement (FID) assay, which employs TO, a light-up fluorescent probe that binds to GQ structures [42]. Candidate ligands can competitively displace TO, allowing for the quantification of their relative affinity. PDS, a well-known specific GQ binder [43], was used as a positive control. The results of the FID assay showed that 5 of the 15 drugs effectively displaced TO from the TERRA GQ structure (Fig. 1A). These drugs are fedratinib, netarsudil, osimertinib, pranlukast, and quercetin (Supplementary Table S1). The concentration required to displace 50% of TO (DC_50_), thus causing a 50% reduction in fluorescence signal (Supplementary Fig. S1), suggests that these compounds bind to TERRA GQ with good affinity, although lower than that of PDS (Table 1), with DC_50_ values ranging from 4.3 to 7.1 μM.

**Table 1.** Ligand DC_50_ values for TERRA GQ determined using the G4-FID assay

Drug	DC_50_ (µM)^a^
Pyridostatin ^b^	0.7
Fedratinib	7.1
Netarsudil	5.5
Osimertinib	4.3
Pranlukast	4.3
Quercetin	6.4

a Ligand concentration required for 50% displacement of TO from the GQ-forming RNA. The error in DC_50_ values is ±5%.

b Positive control.

### TERRA ligands stabilize TERRA at telomeres

Since most GQ binders can interact with both DNA and RNA GQ structures, the specific effect of RNA GQ stabilization at telomeres remains unclear. Additionally, the five drugs selected by the *in vitro* screening have other targets and mechanisms of action. Therefore, to distinguish TERRA-specific downstream biological effects, we focused on those molecules with well-defined specific cytoplasmic targets. Netarsudil, fedratinib, and osimertinib—specific inhibitors of ROCK1, JAK and EGFR, respectively—are FDA-approved antiproliferative drugs with similar IC_50_ values in both HeLa and U2OS cells, whereas quercetin and pranlukast do not show any effect on cancer cells viability and proliferation (Supplementary Fig. S2A). For this reason, fedratinib, netarsudil, and osimertinib were chosen for further investigation. Of note, differently from many other ligands binding DNA GQs, the three compounds showed only modest or no effects on DNA damage response. This was observed both at the level of telomeric damage, measured as the number of TIFs (telomere’s dysfunction-induced foci) per nucleus, (Supplementary Fig. S2B and C) and in terms of global γH2AX phosphorylation, a marker for DNA damage response (Supplementary Fig. S2D), when compared to RHPS4, a well-characterized DNA GQ ligand [44].

To understand whether GQ stabilization could affect TERRA abundance and localization, U2OS cell line, which exhibits high levels of TERRA expression, was treated with IC_50_ doses of the selected compounds. The well-characterized TERRA GQ binder BPBA [43] was used as a reference treatment. A TERRA-specific RNA–FISH assay was performed in combination with an anti-TRF2 immunofluorescence staining to measure the localization of TERRA at telomeres (Fig. 1B). The mean number of colocalizations between the TERRA RNA–FISH probe and the anti-TRF2 antibody signals per nucleus was scored by the MatCol software [44], showing a marked increase of colocalization in treated conditions (Fig. 1C). The abundance of TERRAs, analyzed by real-time qPCR in the same cells, showed that some of the ligands (fedratinib, osimertinib, and BPBA) increased specific TERRA accumulation, as also previously reported [23] (Fig. 1D). To get a more comprehensive view on the effect of ligands on whole TERRA levels, we performed a northern blot, which revealed a modest overall upregulation of TERRA by the compounds fedratinib, osimertinib, and BPBA (Supplementary Fig. S3A and B).

### TERRA GQ ligands interact with telomeric DNA:RNA hybrids both *in vivo and in vitro*

**Figure 2.**
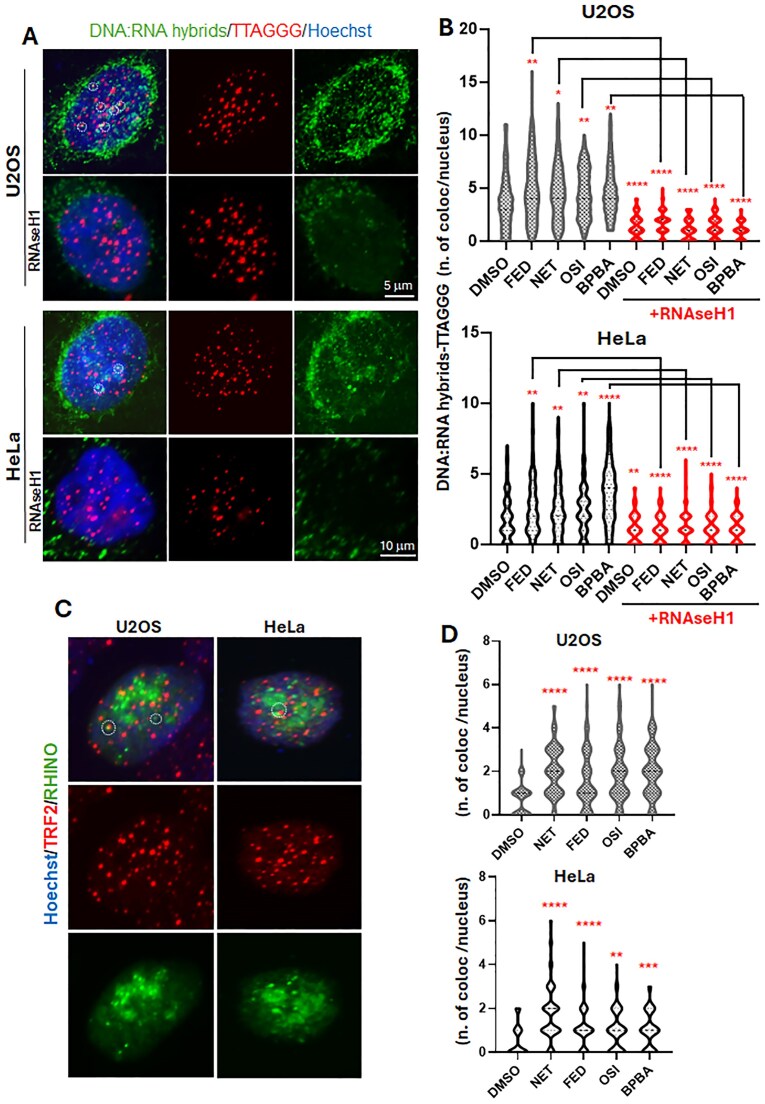
*In cellulo* detection of DNA:RNA hybrids. (**A**) Representative images of DNA:RNA hybrids staining and colocalization at telomeres (60× magnification) in vehicle-treated and RNAseH1-treated samples. Images represent the MIP of 60–80 µm Z-stacks (z. step 0.60 µm). Cells were incubated with the anti-DNA:RNA hybrid S9.6 antibody followed by hybridization with a Cy3-labeled telomeric PNA probe. (**B**) Quantification of colocalizations between anti-DNA:RNA hybrids spots and telomeric spots. The graph reports the number of colocalizations per nucleus. Three independent experiments have been performed, at least 50 nuclei per sample were scored and results were pulled. Statistical significance was calculated using the Kruskal–Wallis test (*P < .05; **P < .01; ***P < .001; ^****^P < .0001). (**C**) Representative images of RHINO-transfected HeLa and U2OS and colocalization of GFP signal at telomeres (60× magnification). Images represent the MIP of 60–80 µm Z-stacks (z. step 0.60 µm). (**D**) Quantification of colocalizations between GFP spots and telomeric spots. The graph reports the number of colocalizations per nucleus. Two independent experiments have been performed, and results were pulled. *N* = 100. Statistical significance was calculated using the Mann–Whitney test (*P < .05; **P < .01; ***P < .001; ^****^P < .0001).

TERRA molecules are known to bind to the complementary telomeric DNA, forming DNA:RNA hybrids that generate an R-loop on the G-rich displaced DNA strand [5]. These structures contribute to increased replication stress at telomeres, a hallmark of ALT cells, and are known to trigger ALT recombination [16].To analyze the effect of TERRA ligands on RNA:DNA hybrids at telomeres, we performed an IF/FISH assay, combining immunofluorescence staining using the anti-DNA:RNA hybrid antibody with a telomeric-DNA FISH (Fig. 2A) in both ALT- (U2OS) and telomerease-positive (HeLa) cells, the latter expressing lower levels of TERRA. In HeLa cell line, the effect of the ligands on TERRA abundance was checked by qPCR and northern blot (Supplementary Fig. S3A–C) revealing a similar trend compared to U2OS upon exposure to ligands. Quantification revealed an increased number of colocalizations upon treatment with all the ligands (Fig. 2B). To assess the specificity of signals of S9.6 antibody, we performed the IF analysis after treatment with RNAseH1, which resulted in a marked reduction of both S9.6 signal and colocalizations in all the conditions. Nevertheless, some perinuclear and nuclear staining resisted the treatment. To confirm the above data with a more reliable experimental tool to visualize R-loops, both HeLa and U2OS cells were transfected with the RHINO plasmid [26], which encodes for the RNAseH1 binding domain fused to a green fluorescent protein (GFP) and can be used as an *in vivo* marker for R-loops. Next, we performed an IF assay with a telomere marker (TRF2) (Fig. 2C). By quantifying the number of colocalizing spots, we observed that TERRA GQ ligands significantly increased R-loops at telomeres in both HeLa and U2OS cells (Fig. 2D). This observation, along with the increased TERRA signal at telomeres observed in the RNA FISH assay, suggests that TERRA GQ ligands may stabilize TERRA binding to telomeric DNA.

It has been shown that G-rich RNA transcripts can intermix with the nontemplate DNA strand during transcription, forming another species, the DNA:RNA hybrid G-quadruplex (HGQ) [45–47]. This raises the possibility that TERRA may contribute to the population of GQ structures through intermolecular interaction with the G-rich telomeric strand, forming an HGQ whose biological significance remains elusive. Also, growing evidence suggests that HGQ structures can be involved in the mode of binding of TERRA to the G-rich 3′ telomeric overhang [5, 6, 48–50]. Therefore, we investigated the ability of TERRA GQ ligands to stabilize telomeric HGQ structures that potentially forms at telomeric R-loops.

**Figure 3.**
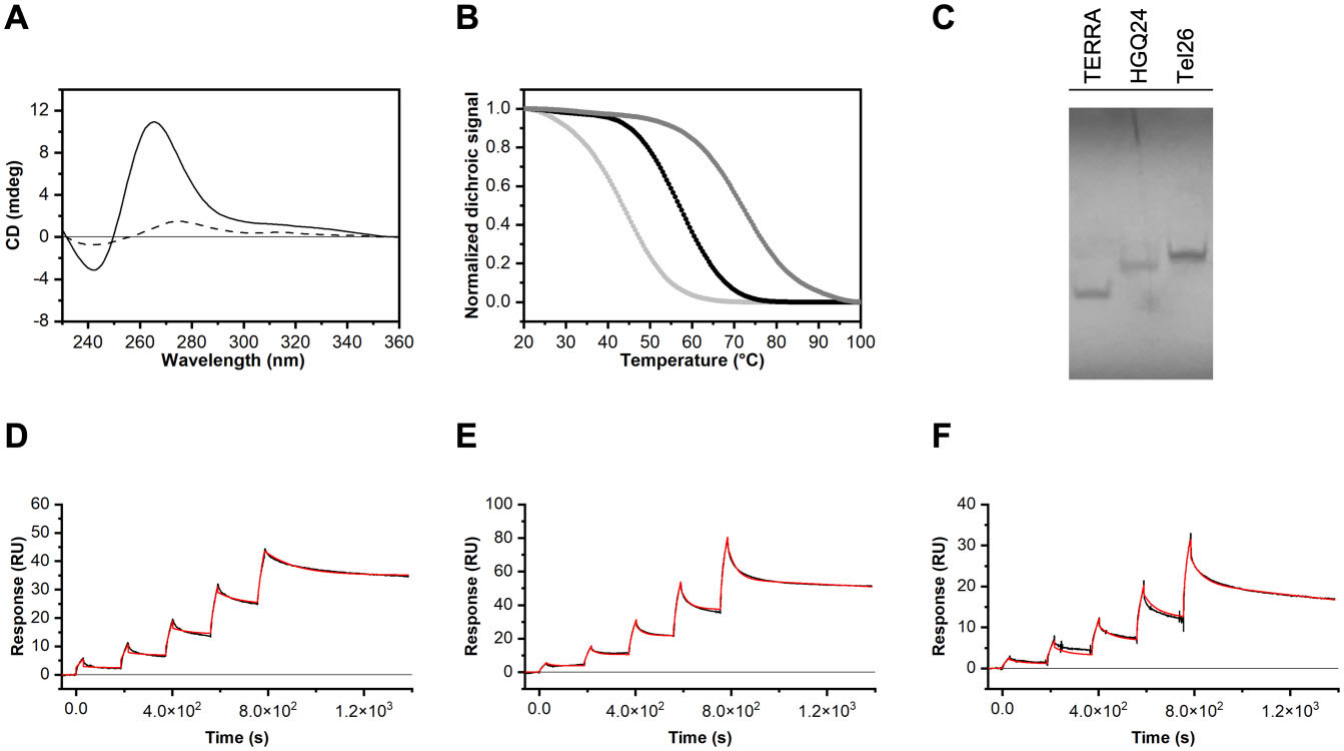
*In vitro* characterization of HGQ24. (**A**) CD spectra of HGQ24 at 20 and 100°C (solid and dashed lines, respectively). (**B**) Normalized CD melting profile of HGQ24, TERRA, and Tel26 GQs (black, dark gray, and light gray, respectively) recorded at 1.0°C/min, by monitoring CD signal at 290 nm for Tel26 and 264 nm for HGQ24 and TERRA GQs. (**C**) Native PAGE (20% polyacrylamide gel, 29:1 acrylamide:bisacrylamide) run at 4°C and 120 V for 2 h in 1× TBE buffer. Oligonucleotide samples were prepared at 20 μM concentration in 5 mM phosphate buffer (pH 7.0) supplemented with 20 mM KCl. Glycerol was added (10% final) to facilitate sample loading in the wells. Bands were visualized by UV shadowing at 254 nm. Time evolution SPR sensorgrams obtained at 25°C by injections of various concentrations (from 0.062 to 1 μM) of (**D**) HGQ24, (**E**) TERRA, and (**F**) Tel26 GQs on the chip-immobilized BG4. The sensorgrams are shown as black lines and their respective fits as red lines.

To assess the binding of selected drugs to telomeric HGQ *in vitro*, we used a model HGQ consisting of a 24-nt long chimeric DNA–RNA oligonucleotide with half DNA and half RNA telomeric repeats [TAGGGTTAGGG*UUAGGGUUAGGGU* (RNA residues are in italics), referred to as HGQ24]. This model has the advantage to avoid the formation of mixed structures, typically observed in preparations combining DNA and RNA sequences, in which the hybrid form is only partially achieved [48–50]. We first verified the ability of HGQ24 to form a GQ structure, assessed its conformation, and determined its thermal stability through circular dichroism (CD) experiments. The CD spectrum of HGQ24 revealed that it adopts a parallel GQ conformation, as evidenced by the positive band around 264 nm and the negative one at 240 nm (Fig. 3A). This is consistent with a previous study showing that hybrid DNA:RNA G-rich sequences typically adopt parallel GQ topologies [51]. A melting temperature (*T*_m_) of 58.5°C was determined for HGQ24, indicating the formation of a stable GQ structure (Fig. 3B), with thermal stability intermediate between that of TERRA and Tel26 GQs. Moreover, PAGE analysis showed that HGQ24 migrates with intermediate mobility between GQs formed by TERRA and the telomeric DNA (Tel26) (Fig. 3C). Collectively, these results suggest that HGQ24 forms a stable monomolecular GQ structure with a parallel conformation.

We also assessed whether the BG4 antibody, commonly used for detecting GQ formation [52], can recognize HGQ24. Using SPR experiments, we analyzed this interaction and evaluated the binding affinity of BG4 for HGQ in comparison to the GQs formed by Tel26 DNA and TERRA RNA (TERRA) sequences. SPR sensorgrams, obtained by injecting increasing concentrations of GQ-forming sequences over a BG4-functionalized sensor-chip surface, showed a response proportional to analyte concentration, demonstrating the specificity of GQ-antibody interactions (Fig. 3D–F). The sensorgrams were fitted to a 1:1 binding model, enabling the determination of the kinetic rate constants, *k*_on_ and *k*_off_, and the calculation of the equilibrium dissociation constants (*K*_d_) as *k*_off_/*k*_on_ (Table 2). The results revealed that BG4 binds to HGQ24 and TERRA (both with parallel GQ topologies) with similar affinity, while binding with two-fold lower affinity to Tel26, which adopts a [3+1]-hybrid GQ topology (see CD spectra in Supplementary Fig. S4A and B). Overall, these findings indicated that BG4 binds to HGQ24 as efficiently as it binds to DNA or RNA GQs, supporting the formation of the DNA:RNA HGQ structure.

**Table 2.** Kinetic parameters and equilibrium dissociation constants for the interaction of HGQ24, TERRA, and Tel26 GQs with the BG4 antibody, determined by SPR

Analyte	*k*_on _× 10^4^ (M^−1^ s^−1^)^a^	*k*_off_ × 10^−4^ (s^−1^)^a^	*K*_d_ (nM)^b^
HGQ24	2.84	3.20	11.3
TERRA	2.89	3.73	12.9
Tel26	2.53	6.34	25.1

a Errors were within 5%.

b Errors were within 10%.

Next, we examined fedratinib, netarsudil, and osimertinib binding to HGQ24 employing the FID assay initially used for ligand screening on TERRA GQ. The assay was extended to include Tel26 GQ for comparison (Supplementary Fig. S4C and D, and Table 3). The three drugs also showed displacement activity on HGQ24, although to a lesser extent than TERRA. Specifically, netarsudil and osimertinib showed DC_50_ values of 6.2 and 7.7 μM, respectively, which were slightly higher than those observed for TERRA GQ (5.5 and 4.3 μM, respectively; Table 1). In contrast, fedratinib exhibited significantly weaker displacement on HGQ24, with a DC_50_ value of 35.0 μM. For Tel26, only netarsudil and osimertinib were able to displace TO from the GQ, with DC_50_ values of 5.9 and 9.8 μM, respectively. To further characterize ligand binding to HGQ24 and Tel26, fluorescence-based titration experiments were performed using fluorophore-labeled GQs (Cy5.5-HGQ24 and Cy5.5-Tel26). Upon ligand addition, a dose-dependent decrease in Cy5.5 fluorescence emission was observed (Supplementary Fig. S5), enabling the determination of *K*_d_ under isothermal conditions. The results (Table 3) showed that osimertinib binds both GQ structures with higher affinity than fedratinib, exhibiting a slight preference for HGQ24 [*K*_d_ = 10.8 (±2.5) μM] over Tel26 [*K*_d_ = 13.4 (±2.1) μM], consistent with TO displacement assay results. Similarly, fedratinib showed preferential binding to HGQ24 compared to Tel26, with *K*_d_ values of 30 (±7) μM and 76 (±4) μM, respectively, also in agreement with the TO data. In contrast, accurate *K*_d_ determination for netarsudil was not possible due to complex titration patterns obtained with both GQs, resembling previously reported interactions for well-known GQ ligands such as PhenDC3 [32], and suggesting multi-step binding processes and/or non-specific quenching at high concentrations.

**Table 3.** Ligand DC_50_ and *K*_d_ values for HGQ24 and Tel26 GQs determined with the FID assay, and fluorescence titrations, respectively

	DC_50_ (µM)^a^		*K*_d_ (µM)	
Drug	HGQ24	Tel26	HGQ24	Tel26
Fedratinib	35.0	n.d.^b^	30 (±7)	76 (±4)
Netarsudil	6.2	5.9	n.a.^c^	n.a.
Osimertinib	7.7	9.8	10.8 (±2.5)	13.4 (±2.1)

a Ligand concentration required for 50% displacement of TO from GQ structures. The error in DC_50_ values is ±5%.

b n.d.= no significant displacement detected.

c n.a.= not available.

To evaluate the effects of these drugs on GQ stability *in vitro*, CD melting experiments were performed. Indeed, ligand binding to a GQ typically stabilizes the structure, resulting in an increase in the melting temperature (*T*_m_). This stabilization is assessed by monitoring the *T*_m_ in the presence and in the absence of the ligand (Supplementary Fig. S6). As summarized in Table 4, osimertinib significantly stabilized all GQs, regardless of their topology or composition (DNA, RNA, or hybrid), with the strongest effect observed on HGQ24. Fedratinib exhibited a marked stabilizing effect on HGQ24, followed by Tel26, but had only a minor effect on TERRA GQ, while netarsudil significantly stabilized HGQ24, showing a clear preference over TERRA and Tel26.

**Table 4.** Drug-induced thermal stabilization of HGQ24, Tel26, and TERRA GQ structures measured by CD melting experiments, and of F-Tel21-T GQ in the absence and presence of large excesses d26 duplex, measured by FRET melting experiments

	$\Delta $ *T*_m_ (°C)^a^					
	CD			FRET		
Drug	HGQ24	Tel26	TERRA	F-Tel21-T	F-Tel21-T/ds26 (1:25)	F-Tel21-T/ds26 (1:50)
Fedratinib	10.6 (±0.2)	7.8 (±0.2)	3.0 (±0.2)	12.1 (±0.5)	10.5 (±0.2)	11.0 (±0.3)
Netarsudil	7.5 (±0.2)	4.3 (±0.2)	4.0 (±0.5)	8.4 (±0.4)	6.8 (±0.3)	6.2 (±0.3)
Osimertinib	14.2 (±0.2)	8.5 (±0.2)	8.0 (±0.2)	8.5 (±0.3)	8.7 (±0.3)	8.2 (±0.2)

a Δ*T*_m_ represents the difference in melting temperature [Δ*T*_m_ = *T*_m_ (GQ + 10 drug equiv) − *T*_m_ (GQ)]. The *T*_m_ values of GQ alone are: HGQ24 = 58.5 (±0.1)°C, Tel26 = 43.3 (±0.1)°C, TERRA = 72.2 (±0.1)°C, and F-Tel21-T = 50.6 (±0.1)°C.

The apparent discrepancy between the binding results (TO displacement and fluorescence titration experiments) and thermal stabilization data may arise from different drug-binding mechanisms to GQ structures. For instance, ligand binding may not necessarily directly compete with TO, underscoring the complexity of ligand-GQ interactions. Additionally, the lower thermal stabilization effects observed for TERRA might be due to its inherently higher thermal stability, emphasizing the importance of integrating data from multiple techniques for a comprehensive analysis.

Finally, to assess the selectivity of these compounds for GQ over the canonical BDNA, competition FRET melting experiments were performed using a telomeric GQ-forming oligonucleotide labelled with FAM and TAMRA (F-Tel21-T) and a large excess of unlabeled duplex DNA (ds26) as a competitor (Supplementary Fig. S7). As summarized in Table 4, the GQ-stabilizing effects of fedratinib, netarsudil, and osimertinib were only marginally reduced in the presence of the competitor DNA, indicating a high degree of selectivity for GQ structures over duplex DNA.

Taken together, these findings suggest that TERRA molecules can bind to telomeric DNA not only by binding the C-rich DNA strand through canonical Watson-Crick pairing but also by binding the G-rich strand through the formation of DNA:RNA hybrid GQs. Interestingly, some TERRA GQ ligands appear even more effective in stabilizing these hybrid GQ structures than their homologous counterparts.

### TERRA ligands induce telomeric defects and ALT phenotype

**Figure 4.**
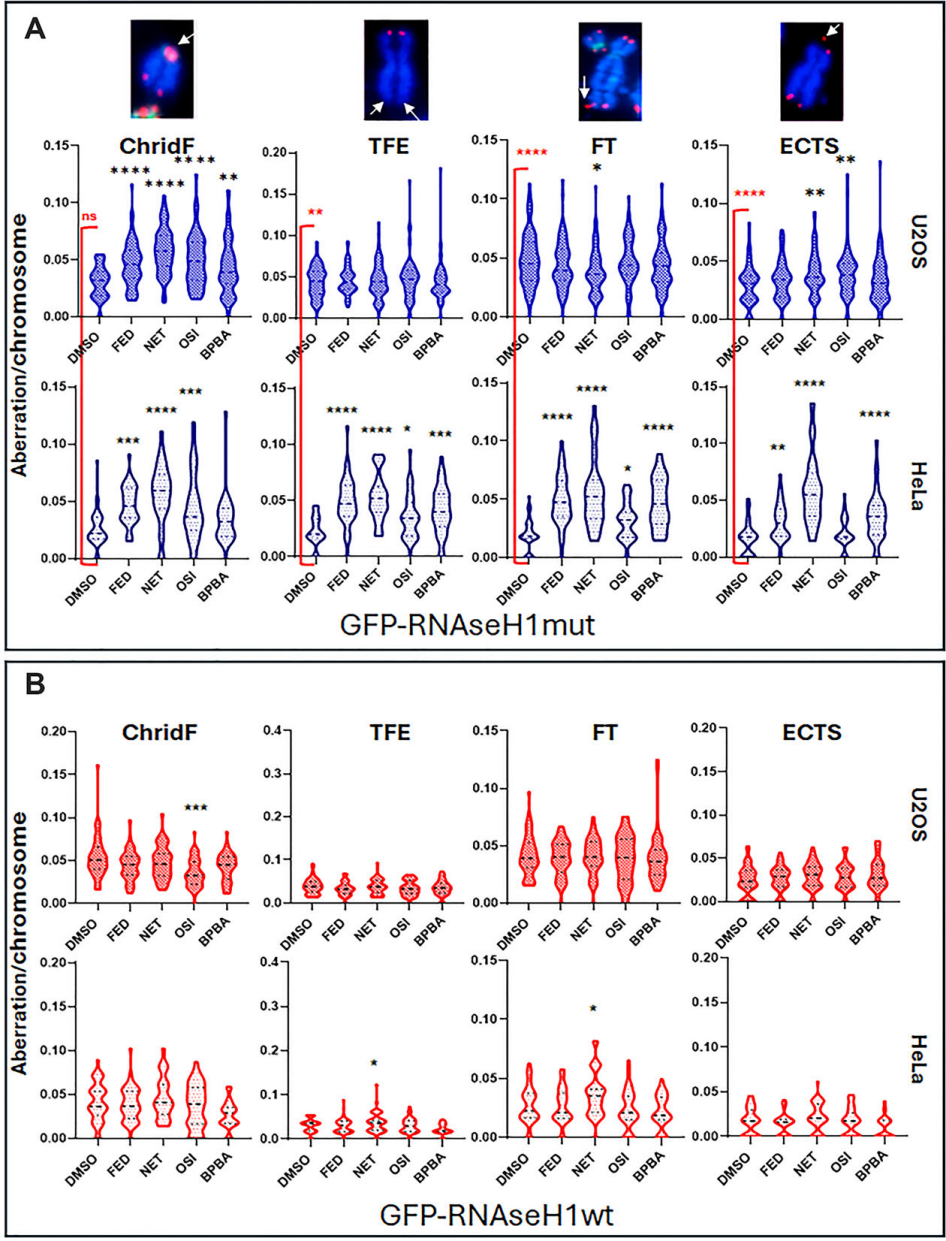
Effects of TERRA GQ ligands on mitotic telomeric defects. HeLa and U2OS cells were transfected with (**A**) GFP-RNAseH1 mut or (**B**) GFP-RNAseH1 wild-type constructs. Then, cells were treated for 48 h with the IC_50_ concentrations of the indicated compounds and were arrested in mitosis, spread onto glass slides, and processed for FISH using a Cy3-labeled telomeric probe (Cy3-TelC) and FITC-labeled pancentromeric probe. Representative images of various telomeric aberrations identified are shown in the upper panels (100× magnification). The graphs present the quantified number of telomeric aberrations per chromosome observed in each metaphase. Two independent experiments were performed, and results were pooled. *N* > 60. Statistical significance was calculated using the Kruskal–Wallis test. *P > .05; **P > .01; ***P > .001; ^****^P > .0001.

The formation of DNA:RNA hybrids is a significant source of replication stress that increase the likelihood of replication/transcription fork collisions [53]. Consequently, the induction of R-loops at telomeres by TERRA GQ stabilization suggests that cell treatment with these ligands could exacerbate telomeric defects caused by the resolution of stalled replication forks. Indeed, ALT-positive cells, which experience high replication stress at telomeres, accumulate telomeric defects that can be analyzed using a telo-FISH assay on metaphase spreads. Specifically, fragile telomeres (FT), characterized by a double telomeric signal on a single chromatid, are directly associated with replication stress at telomeres. Telomere free ends (TFE), characterized by the absence of telomeric signals at chromosome extremity, and extra chromosomal telomeric signals (ECTS), identified as telomeric spots outside the chromosome, can result from breakage in subtelomeric or telomeric regions. Additionally, a recent study suggests that TERRA may influence the disjunction of sister chromatids during mitosis, potentially affecting the frequency of chromatidic fusions (ChridF) [54]. To investigate the impact of treatment with TERRA GQ ligands on these telomeric defects, we performed a telo-FISH assay on metaphase spreads from HeLa and U2OS cells treated with TERRA GQ ligands or DMSO and quantified the frequency of the observed phenotypes. As shown in Supplementary Fig. S8, the basal frequencies of FT, TFE, and ECTS are higher in U2OS compared to HeLa cells, which is consistent with the elevated replication stress and the resulting chromosomal rearrangements typically observed in ALT cells. Notably, in both HeLa and U2OS cells, there is a significant increase in the frequency of ChridF, supporting the proposed role of TERRA in sister chromatids disjunction [54]. This finding also suggests that GQ structures may contribute to stabilizing chromatid tethering. A possible mechanism for this could involve a single TERRA molecule bridging the two sister chromatids, either by binding the C-rich strand by sequence complementarity or by interacting with the G-rich strand via an intermolecular HGQ. Interestingly, the overall induction of FT, TFE, and ECTS is significantly greater in HeLa cells, suggesting that TERRA GQ stabilization may trigger a switch toward an “ALT-like” telomeric phenotype. In contrast, in U2OS cells, which already experience higher replication stress and may have activated cellular tolerance mechanisms, ChridF emerges as the most affected phenotype. ChridF frequency increases with all treatments, except BPBA, highlighting the different affinity and binding mode of the ligands. To ascribe the cytogenetic effects of ligands to the stabilization of DNA:RNA hybrids, we repeated the same measurements in U2OS and HeLa cells overexpressing either wild-type or mutant GFP-tagged RNAseH1 (Fig. 4). The cells were transiently transfected with these constructs, and transfection efficiency was assessed by fluorescence microscopy 24 h later, ranging from 82%–85% in U2OS cells to 90%–95% in HeLa cells, depending on the experiment. Then, the cells were processed as described above for metaphase chromosome analysis. The results revealed a significant reduction of cytogenetic effects in all ligand-induced phenotypes in wild-type RnaseH1 overexpressing cells, together with a clear overlap between untransfected and RNAseH1-mutant transfected cells (Supplementary Fig. S8). These findings confirm the involvement of DNA:RNA hybrids in the formation of telomeric defects.

**Figure 5.**
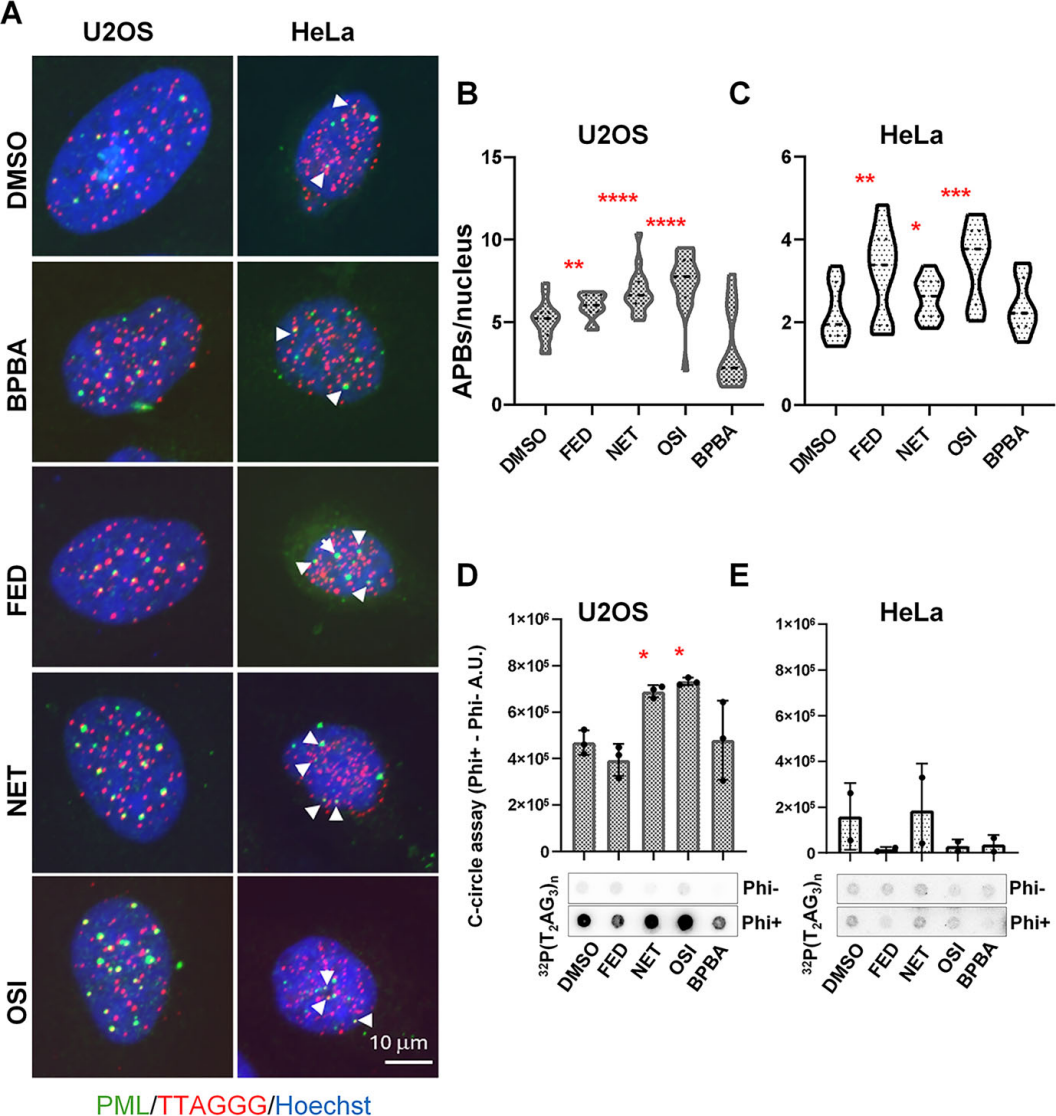
Analysis of ALT activation. (**A**–**C**) APB quantification. U2OS and HeLa cells were treated with the IC_50_ concentration of the indicated compounds for 24 h and analyzed by IF/DNA FISH using an anti-PML antibody and the TelC-Cy3 probe. Representative images at 60× magnification are shown in panel (**A**), displaying the MIP of 60–80 µm Z-stacks (z. step 0.60 µm). The number of APBs, identified by colocalization of red (TelC-Cy3) and green (Cy5-PML) signals, was quantified per nucleus using MatCol software. Quantitative results are reported in graphs (**B, C**). Three independent experiments were performed, and the results were pooled. *N* > 100. The statistical significance was calculated using the Mann–Whitney test (*P < .05; **P < .01; ***P < .001; ^****^P < .0001). (**D, E**): C-circle assay. In U2OS cells treated with the IC_50_ dose of the indicated compounds for 48 h, genomic DNA was extracted, and rolling circle amplification was performed with Phi DNA polymerase. The fold change in telomeric DNA content (normalized to single copy gene determined DNA content) was calculated for Phi-amplified versus nonamplified DNA samples and reported as histograms. Results represent the mean of three independent experiments (shown as individual points in the graph), with bars denoting SD. Statistical significance was calculated with the paired *t*-test (*P < 0.05).

ALT cells are characterized by the presence of ALT-associated PML bodies (APBs), which are specialized subnuclear structures where homologous recombination enzymes and telomeric proteins colocalize. The presence of APBs indicates active ALT recombination and fluctuations in the number of APB per nucleus provide a direct measure of changes in ALT recombination activity, either increased or impaired. To assess whether GQ stabilization induced by fedratinib, netarsudil, and osimertinib affects ALT activity, we evaluated the impact of these drugs on APB formation using immuno-FISH staining. This technique combines anti-PML antibody labeling with Telo-probe hybridization, enabling quantification of PML and Telo-probe colocalization, which marks the presence of APBs (Fig. 5A). The results clearly demonstrate that all three compounds significantly increased the mean number of APBs per nucleus in both U2OS and HeLa cells (Fig. 5B and C, respectively), indicating an increased frequency of ALT recombination following treatment. However, despite its binding capacity, BPBA did not significantly influence ALT activity (Fig. 5B and C). To further quantify the effect of ligands on ALT activity in U2OS cells, we measured C-circle levels (Fig. 5D and E) [31]. As shown in Fig. 5E, fedratinib and osimeritnib, which showed the most consistent activity on APBs induction, robustly promoted C-circle formation in U2OS. Conversely, in HeLa cells, which express very low levels of C-circles, even if APBs are increased, we cannot detect an increase of C-circles.

**Figure 6.**
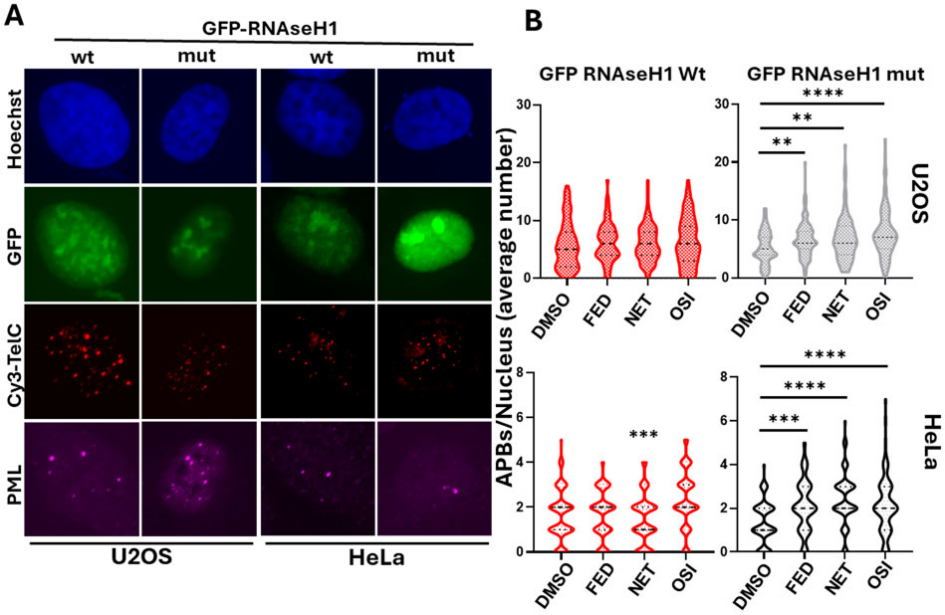
Analysis of APBs in the presence of RNAseH1 overexpression. U2OS cells were transfected with expression vectors encoding GFP along with wild-type or mutated RNAseH1. After 24 h, cells were treated with the IC_50_ concentration of the indicated compounds for an additional 24 h and then processed for IF/DNA FISH. This involved staining with an anti-PML antibody, followed by a Cy5-conjugated secondary anti goat antibody, and the TelC-Cy3 probe. Representative images at 60× magnification are presented in panel (**A**), showing MIP of 60–80 µm Z-stacks (z. step 0.60 µm). To quantify APBs, images were processed as follows: the green (GFP) channel was removed, and the purple (Cy5-PML) channel was converted to a green pseudo color, to obtain RGB images. APBs (identified as colocalized red and green signals) were quantified per nucleus using MatCol software. The results are reported in the graphs in panel (**B**). Three independent experiments were performed, with pooled results. *N* > 100. Statistical significance was calculated with the Mann–Whitney test (*P < .05; **P < .01; ***P < .001; ^****^P < .0001)

Finally, to confirm that the effectiveness of ligands in modulating APBs is directly linked to TERRA stabilization, we transfected U2OS and HeLa cells with plasmid vectors expressing GFP-tagged (to mark transfected cells) and either wild-type RNAse H1 or mutant form (D210) (Fig. 6A). RNAse H1 overexpression enhances the degradation of RNA:DNA hybrids and should mitigate effects directly attributable to the formation and/or stabilization of these hybrids. We scored the number of APBs per nucleus in treated and untreated GFP-positive cells (Fig. 6B), showing an impairment of APBs accumulation upon ligand treatments, in presence of RNAseH1 in both HeLa and U2OS. In contrast, the catalytically inactive RNAse H1 mutant did not have this effect.

## Discussion

Through LBVS and experimental validation, we identified FDA-approved drugs capable of binding and stabilizing TERRA GQs both *in vitro* and *in cellulo*: fedratinib, netarsudil, and osimertinib. These ligands were shown to increase TERRA’s association with telomeric DNA, promoting DNA:RNA hybrid (R-loop) formation, which leads to various telomeric abnormalities, including FT, TFE, and ECTS, features that are hallmarks of ALT positive cells.

Importantly, each of these drugs was originally developed to target distinct oncogenic pathways, unrelated to TERRA or GQs. Fedratinib is a selective JAK2 inhibitor approved for the treatment of myelofibrosis, where it functions by disrupting the JAK–STAT signaling pathway, a critical regulator of hematopoietic cell proliferation and survival [55–57]. Netarsudil is a Rho-associated protein kinase (ROCK) inhibitor used in glaucoma therapy to reduce intraocular pressure by affecting cytoskeletal dynamics and aqueous humor outflow [58]. Lastly, osimertinib is a third-generation EGFR tyrosine kinase inhibitor designed to target T790M resistance mutations in nonsmall-cell lung cancer [59, 60]. While these drugs were not originally developed to engage nucleic acid structures, their ability to interact with telomeric GQs highlights an additional mechanism of action that may significantly impact genomic stability.

In the absence of a robust DNA-damage induction as an early event after treatment (Supplementary Fig. S2), we focused our attention on the stabilization of RNA:DNA hybrids as a potential mechanism driving telomeric defects and rearrangements. Notably, unlike other previously characterized GQ ligands, the absence of detectable DNA damage, especially at telomeres, which are enriched in GQ structures, supports the target selectivity of the compounds. This selectivity is consistent with *in vitro* data showing a preferential binding to RNA and DNA:RNA hybrids telomeric GQ. However, we cannot completely exclude the possibility of additional effects related to DNA GQs such as alterations of gene expression. All tested compounds were found to stabilize TERRA at telomeres, as evidenced by an increase in TERRA/telomeric DNA colocalizations (Fig. 1B and C) and elevated levels of R-loops at telomeres (Fig. 2). Although DNA:RNA stabilization at telomeres has been reported to negatively affect TERRA promoter transcription *in vitro* [47], in our experimental settings we observed a mild stabilization. This finding is consistent with the increased number of colocalizing spots and is evident in TERRA FISH staining, as well as in qPCR and northern blot analyses of both HeLa and U2OS cells (Fig. 1B and D, and Supplementary Fig. S3). In this regard, previous studies have also reported increased TERRA levels following GQ stabilization [61] or upon interference with RNA:DNA hybrid suppressors, suggesting a role for these mechanisms in TERRA stabilization [62]. Consistent with these observations, other reports suggest that GQ stabilization may protect RNA from degradation [63]. The involvement of DNA:RNA hybrids in ALT activity is also well established [64]. In our study, stabilization of TERRA GQs by these ligands promoted the formation of ALT-APBs, subnuclear structures that recruit telomeric DNA, proteins, and DNA metabolism enzymes to facilitate homologous recombination-based telomere elongation in ALT cells (Fig. 5A–C). Intriguingly, telomeric abnormalities typical of ALT cells were increased in ALT cells and induced in telomerase-positive cells, suggesting that TERRA GQ stabilization alone may be sufficient to promote a switch toward ALT-like phenotypes, regardless of telomerase activity (Fig. 4A). Notably, both effects were completely reversed by the overexpression of RNAseH1 (Figs 4B and 6), providing compelling evidence that the observed APBs induction by the ligands results from the direct involvement of TERRA stabilization. This is also in agreement with the critical role of TERRA in APBs dynamics, telomeres recombination and, as recently described, in sister telomeres separation during mitosis. Despite TERRA stabilization, and the induction of APBs and telomeric defects in both telomerase positive and ALT background, C-circles accumulation was found hyperactivated only in ALT positive cells by the most effective compounds (Fig. 5D and E). This finding can underlie the activation of different ALT pathways not always resulting in C-circle activation [65].

In conclusion, our findings provide new insights into the interplay between TERRA, GQ structures, and telomeric maintenance mechanisms, positioning TERRA GQ ligands as valuable tools to probe telomere biology. In addition, the ability of these clinically approved agents to induce ALT features and genomic instability highlights a potential risk: their off-target effects on telomeres may contribute to therapy resistance, emphasizing the need for careful evaluation of such consequences in both current treatments and future drug development.

## Supplementary Material

gkaf1300_Supplemental_File

## Data Availability

All data are available at https://gbox.garr.it/garrbox/s/M12L5aYeEofPAAn.
