# Supplementary material for: Modulating TERRA G-quadruplexes with ligands: impact on telomeric DNA:RNA hybrids and ALT mechanisms
Source: Nucleic Acids Res. 2025 Nov 29;53(22):gkaf1300. doi: 10.1093/nar/gkaf1300 (PMC12663085; doi:10.1093/nar/gkaf1300)
Supplement: gkaf1300_Supplemental_File [file gkaf1300_supplemental_file.pdf]

SUPPLEMENTARY MATERIAL

Modulating TERRA G-quadruplexes with ligands: Impact on telomeric DNA:RNA hybrids and ALT mechanisms

Federico Dinoi[1,2]†, Simona Marzano[3,]†, Maria Ilaria Marino[1], Eleonora Vertecchi[1], Carlo Maria D'Angelo[1], Carmen Maresca[4], Eleonora Petti[4], Roberto Dinami[4], Angela Rizzo[4], Annamaria Biroccio[4], Stefano Cacchione[2], Bruno Pagano[3], Erica Salvati[1,*], Jussara Amato[3,*]

[1] *Institute of Molecular Biology and Pathology, National Research Council, Rome, 00185, Italy;*

[2] *Department of Biology and Biotechnology "Charles Darwin", Sapienza University of Rome, Rome, 00185, Italy;*

[3] *Department of Pharmacy, University of Naples Federico II, Naples, 80131, Italy;*

[4] *Translational Oncology Research Unit, IRCCS-Regina Elena National Cancer Institute, Rome, 00144, Italy.*

* To whom correspondence should be addressed. E-mail: erica.salvati@cnr.it.

Correspondence may also be addressed to Jussara Amato. E-mail: jussara.amato@unina.it

† The first two authors should be regarded as Joint First Authors.

Table S1. Name of the selected drugs and their corresponding 2D chemical structures.

**Name**	**Chemical structure**
**Fedratinib**	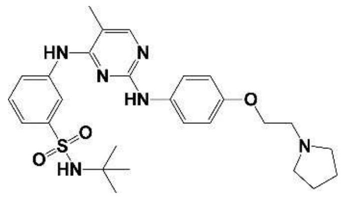
**Netarsudil**	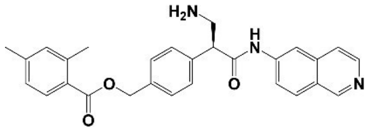
**Osimertinib**	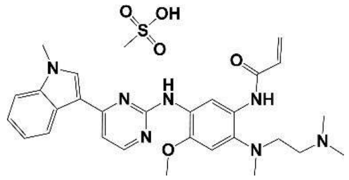
**Pranlukast**	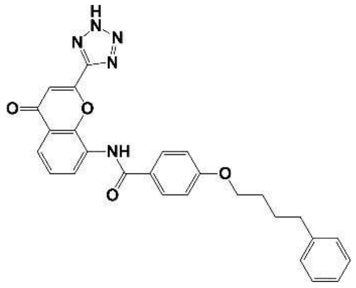
**Quercetin**	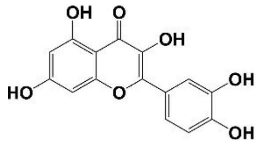

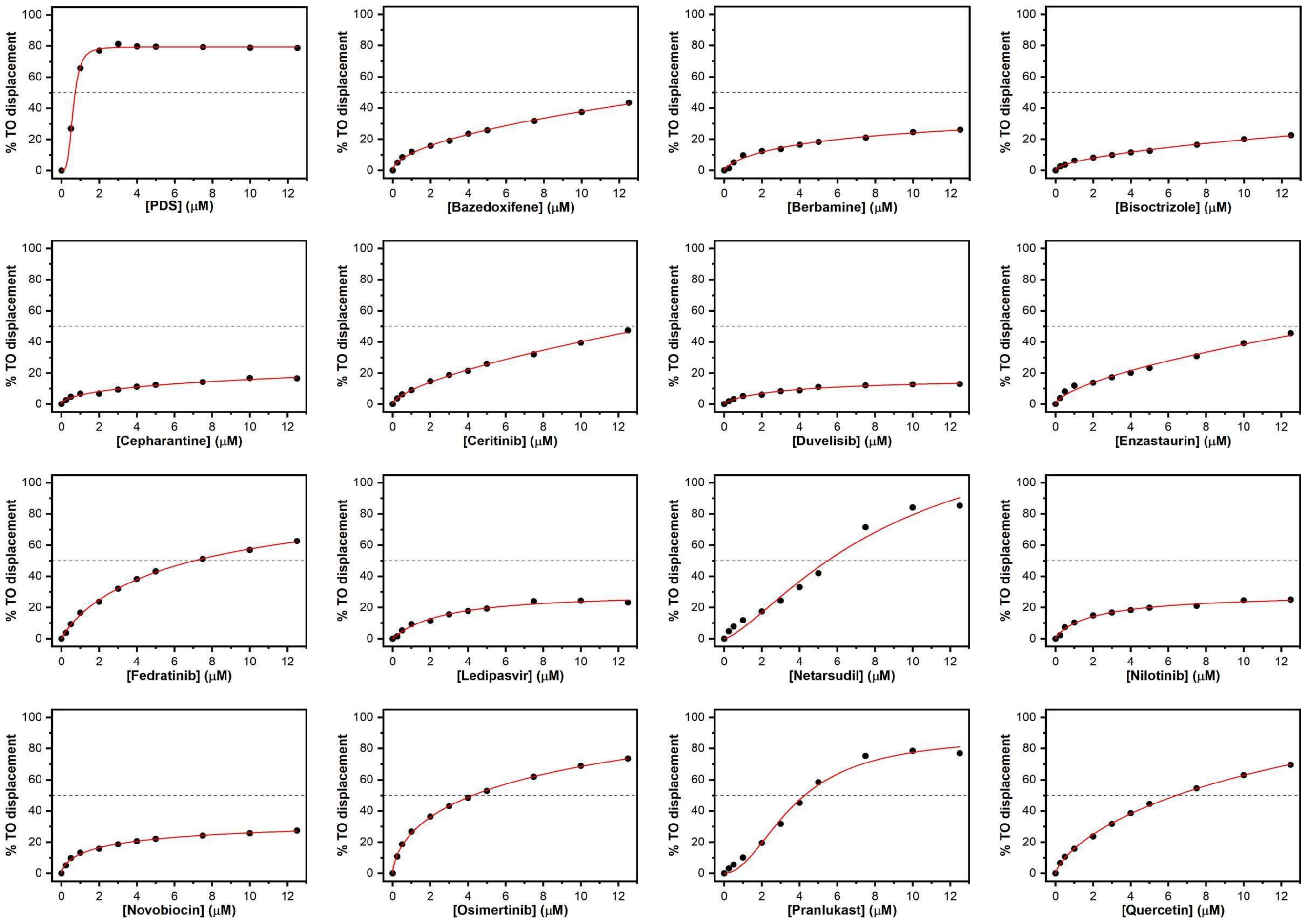

Figure S1. Dose-response curves from fluorescent intercalator displacement (FID) assay of TERRA GQ with pyridostatin (PDS) and the 15 selected drugs.

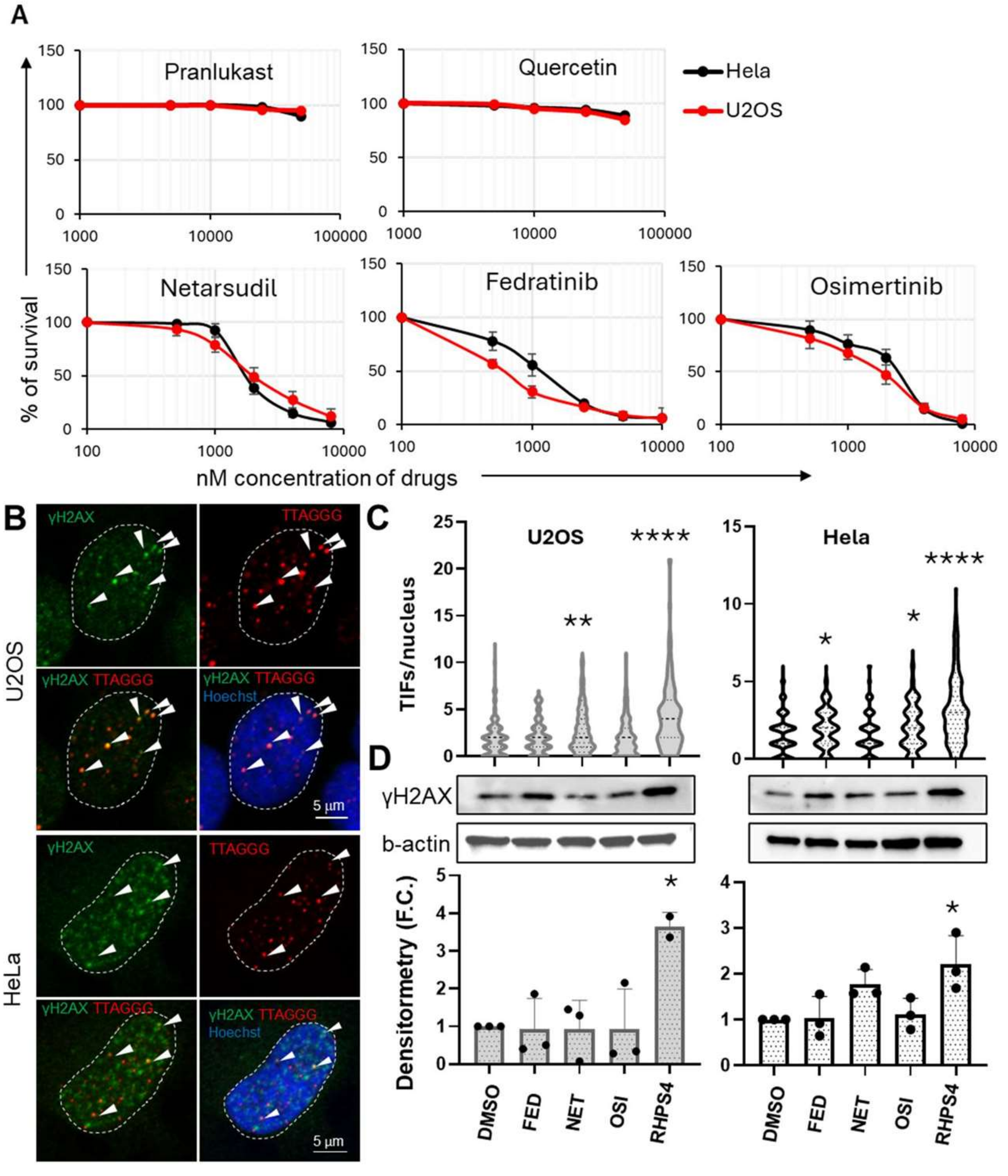

Figure S2. Effects of ligands on cell survival and DNA damage induction. A: Cells were seeded in 24 well plates and exposed to increasing concentrations of compounds for 5 days. Next, cells were stained by crystal violet, resuspended in isopropanol and the absorbance of each sample at OD_{570} was measured by spectrophotometry. The survival of cells was calculated as the percentage of absorbance values of samples vs control and reported in the curves. The mean of three independent experiments is shown. Bars are SD. **B**: Cells were exposed to the IC_{50} of drugs for 24 h and then fixed and processed for immunofluorescence against γH2AX (a marker for DNA damage activation) followed by FISH with Cy3 labelled telomeric probe. Representative images of colocalizations in RHPS4 treated Hela and U2OS at 63x magnification. **C**: The number of colocalizations/nuclei was scored and reported in the graphs. At least 50 nuclei/sample were scored, the experiment was repeated three times, and the results were pulled. Statistical significance of differences was measured with the Kruskal Wallis test. *=P<0.05; **=P<0.001; ****=P<0.0001. **D**: Samples treated as described above were also processed for WB analysis against γH2AX for the measurement of DNA damage activation genome wide. Actin was used as loading control. One out of three independent experiments with similar results is shown. Histograms in the lower panel report the densitometry of WB in triplicates of the experiment. Statistical significance of differences was measured with the Kruskal Wallis test. *=P<0.05. Fedratinib (FED), netarsudil (NET), osimertinib (OSI).

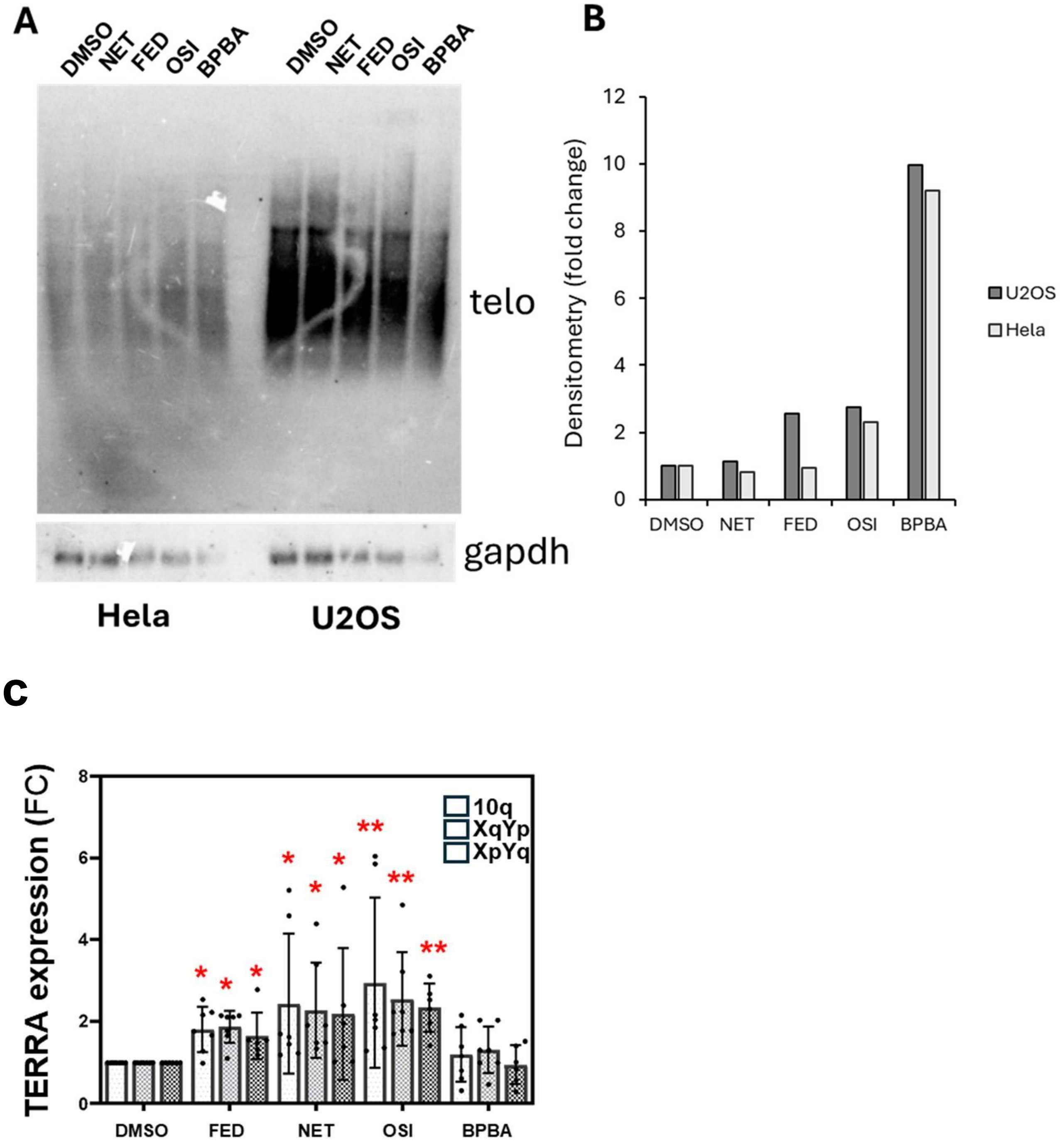

Figure S3. **TERRA Northern blot**. Total RNA extracts from HeLa and U2OS treated with the IC_{50} doses of the indicated compounds for 48 h were processed for Northern blot analysis and hybridized with ^{32}P-radiolabelled teloprobe or gapdh probe as loading control. A single experiment was performed, shown in **A**. Densitometry of the Northern blot (telomeric signal/gapdh signal) is reported in **B**. **C**: TERRA expression in HeLa. Real time qPCR was used to analyze the relative TERRA expression at three different subtelomeric promoters (XYq, XYp, 10q) in HeLa cells treated with the IC_{50} dose of the indicated compounds for 48 h. Results represent the mean of independent experiments (shown as individual points in the graph), with bars indicating standard deviation (SD). Statistical significance was calculated using the Kruskal-Wallis test (* = P<0.05; ** = P<0.01).

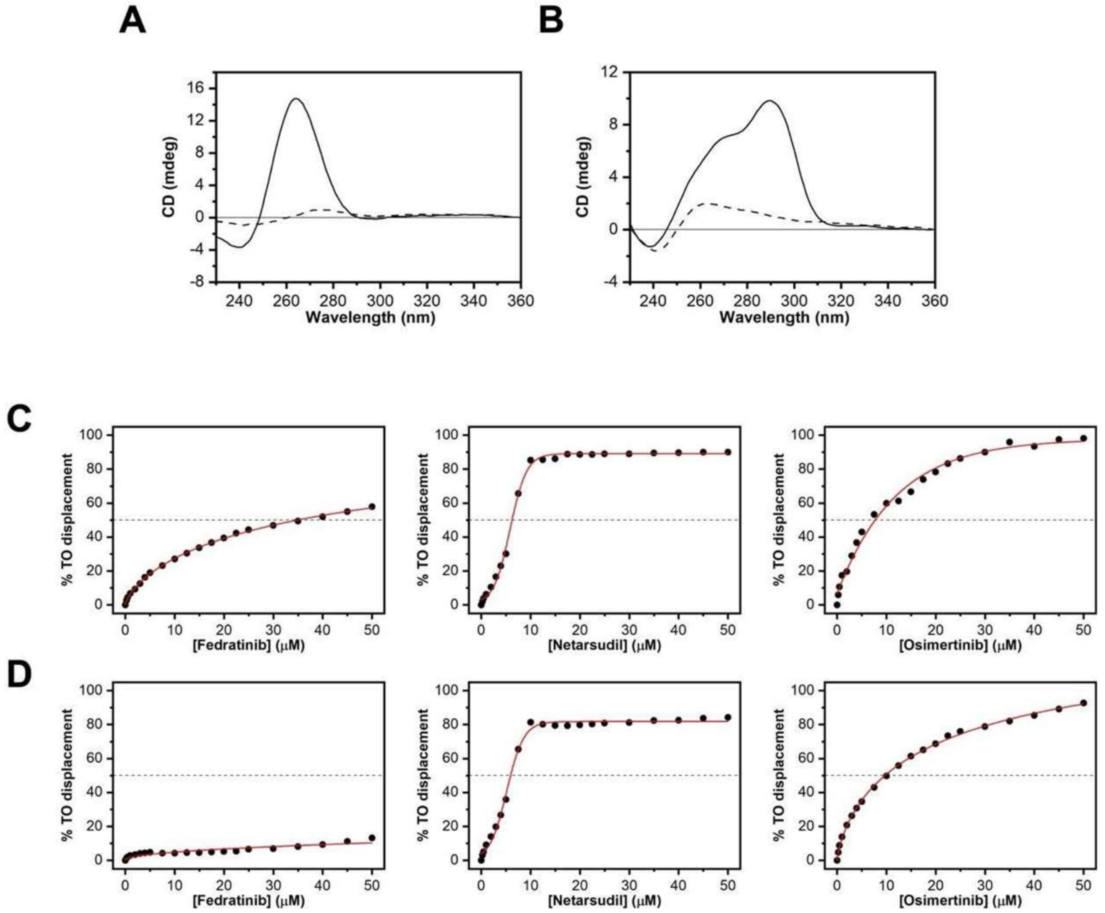

Figure S4. CD spectra at 25 and 100 °C (solid and dashed lines, respectively) of (**A**) TERRA, and (**B**) Tel26 GQs. (**C**, **D**) Representative dose-response curves from FID assay for (**C**) HGQ24 and (**D**) Tel26 GQs with (left) fedratinib, (middle) netarsudil, and (right) osimertinib.

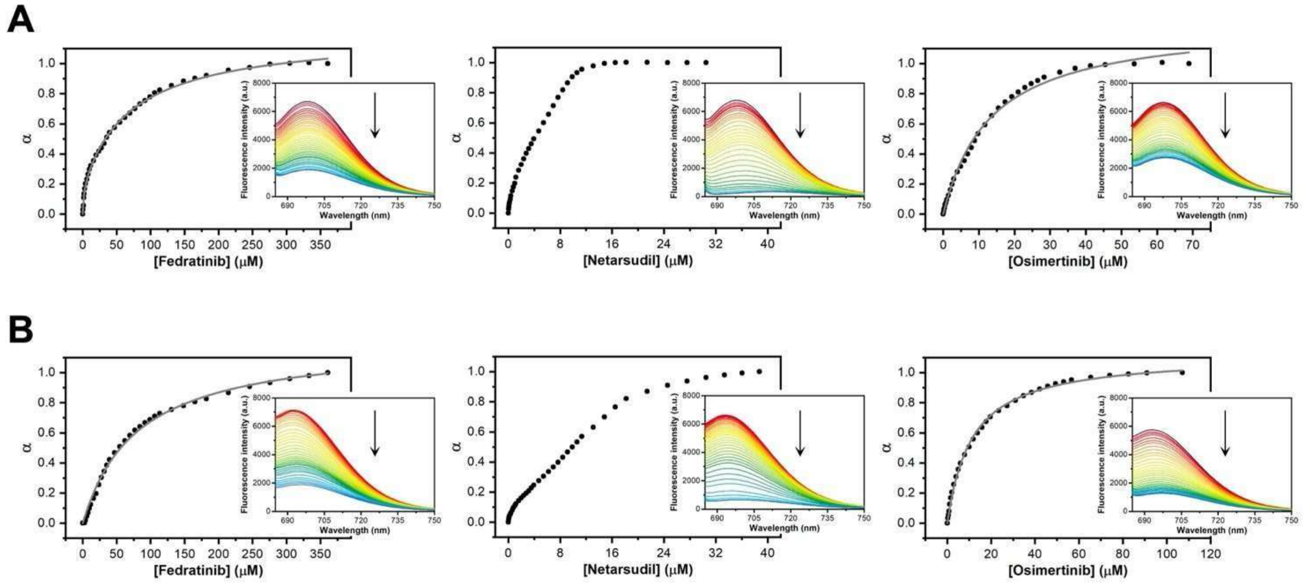

Figure S5. Representative binding isotherms for (**A**) HGQ24 and (**B**) Tel26 GQs with (left) fedratinib, (middle) netarsudil, and (right) osimertinib obtained from fluorescence titration experiments (inserts show the corresponding fluorescence spectra).

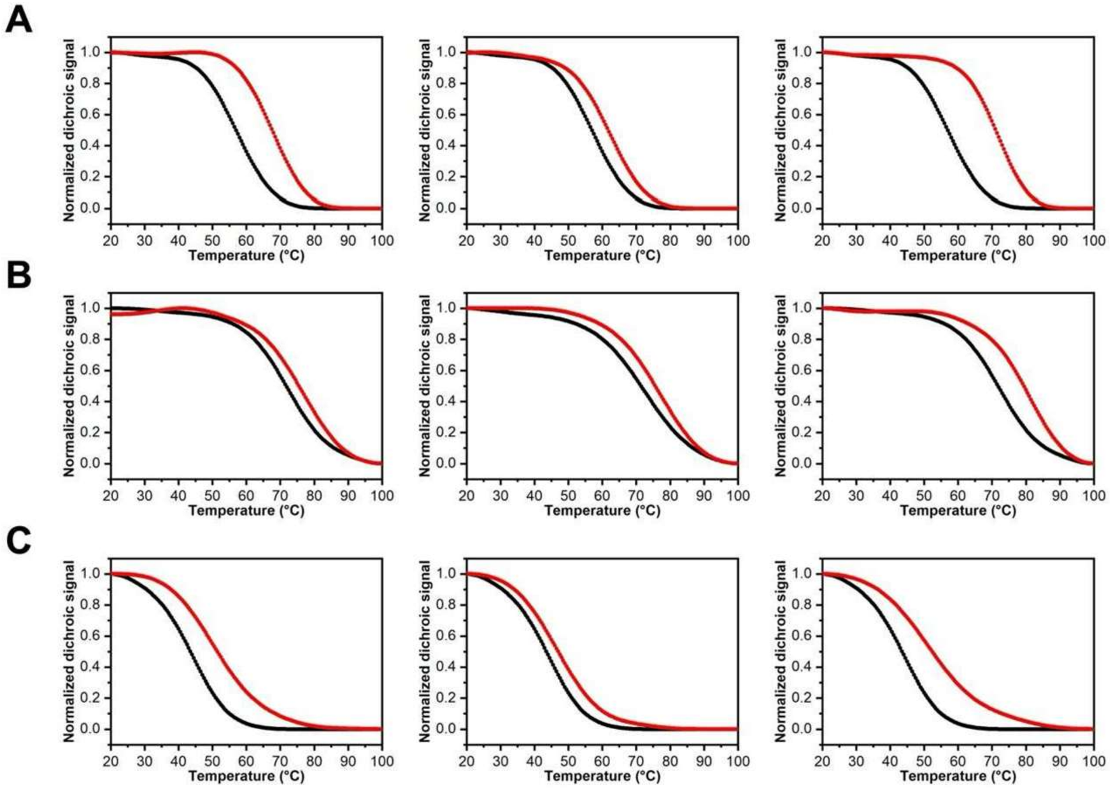

Figure S6. Normalized CD melting curves of (**A**) HGQ24, (**B**) TERRA, and (**C**) Tel26 GQs in the absence (black line) and presence (red line) of 10 mol equiv of (left) fedratinib, (middle) netarsudil, and (right) osimertinib. Experiments were performed at 1 °C/min heating rate, monitoring CD signal at 264 nm for HGQ24 and TERRA, and 289 nm for Tel26.

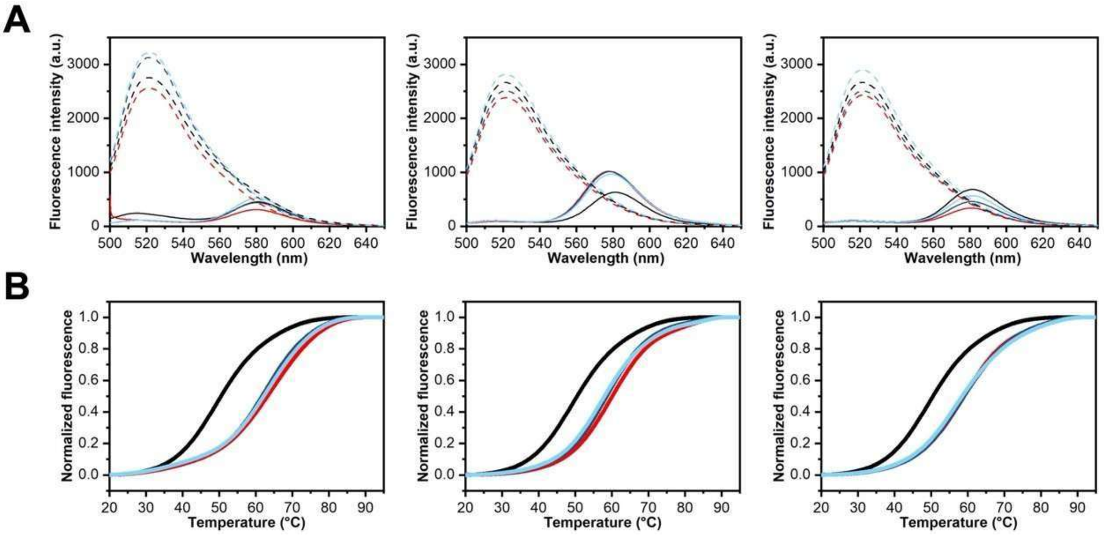

Figure S7. (**A**) Fluorescence emission spectra recorded at 20 °C and 95 °C (solid and dashed lines, respectively) and (**B**) FRET melting experiments for F-Tel21-T (0.2 μM) in the absence (black) and presence (red) of (left) fedratinib, (middle) netarsudil, and (right) osimertinib (2 μM). Experiments in the presence of drugs were also performed by adding an excess of ds26 duplex competitor (5 μM, dark blue; and 10 μM, light blue).

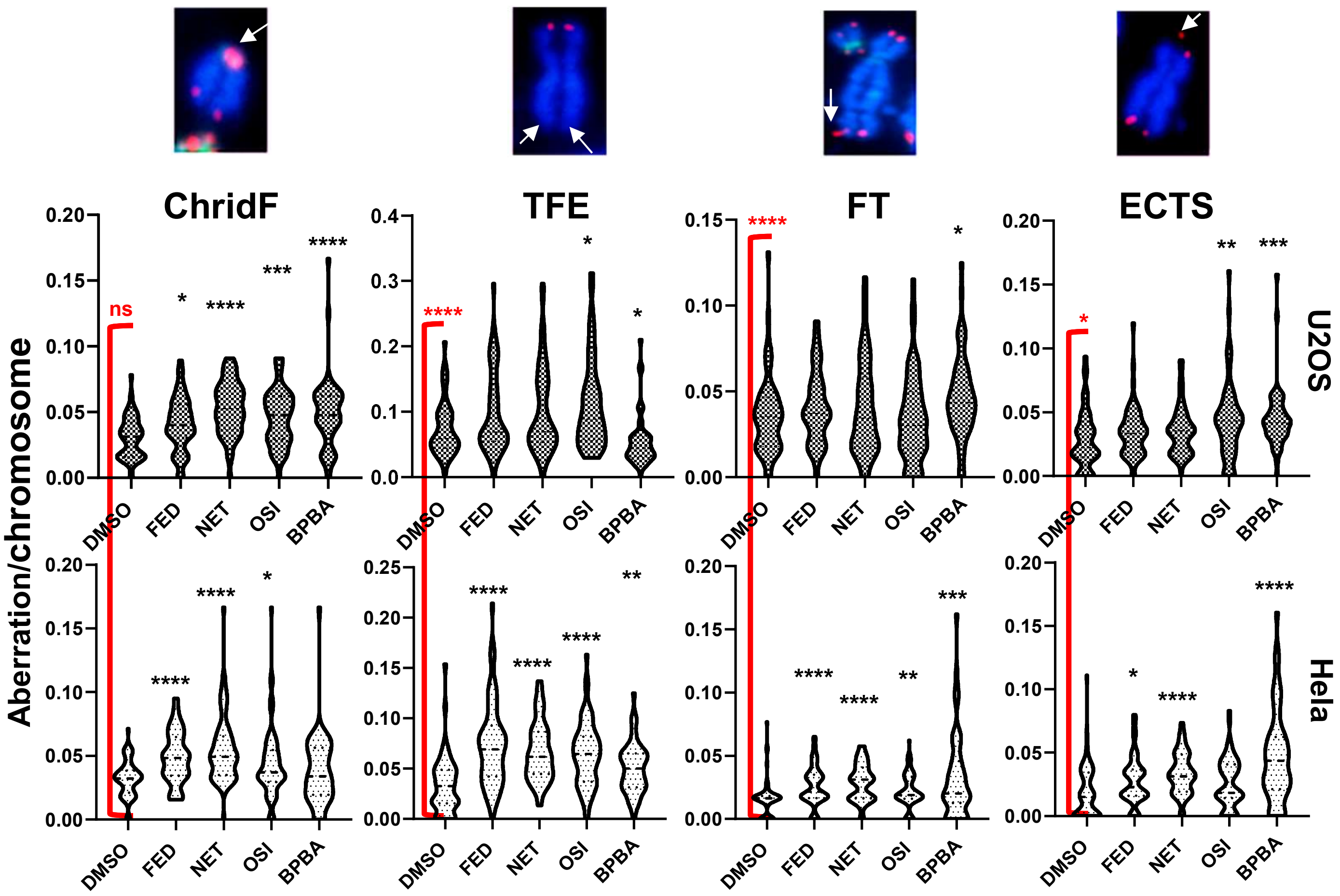

Figure S8. **Effects of TERRA GQ ligands on mitotic telomeric defects.** HeLa and U2OS cells were treated for 48 h with the IC50 concentrations of the indicated compounds, were arrested in mitosis, spread onto glass slides and processed for FISH using a Cy3-labelled telomeric probe (Cy3-TelC) and FITC-labelled pancentromeric probe. Representative images of various telomeric aberrations identified are shown in the upper panels (100× magnification). The graphs present the quantified number of telomeric aberrations per chromosome observed in each metaphase. Two independent experiments were performed, and results were pooled. N>60. Statistical significance was calculated using the Kruskal-Wallis test. * = P>0.05; ** = P>0.01; *** = P>0.001; **** = P>0.0001.
